# Early-stage hepatocellular carcinoma screening in patients with chronic hepatitis B in China: a cost–effectiveness analysis

**DOI:** 10.57264/cer-2023-0146

**Published:** 2024-02-28

**Authors:** Yuemin Nan, Osvaldo Ulises Garay, Xianzhong Lu, Yue Zhang, Li Xie, Zhongyi Niu, Wen Chen

**Affiliations:** 1 Department of Traditional & Western Medical Hepatology, Third Hospital of Hebei Medical University, Shijiazhuang, 050051, China; 2 Roche Diagnostics International, Rotkreuz, ZG, Switzerland; 3 Roche Diagnostics (Shanghai) Co., Ltd, Shanghai, 200335, China; 4 Yidu Cloud (Beijing) Technology Co., Ltd, Beijing, 100083, China; 5 School of Public Health, Fudan University, Shanghai, 200032, China

**Keywords:** chronic hepatitis B, cost–effectiveness analysis, hepatocellular carcinoma, screening

## Abstract

**Aim:**

To evaluate the cost–effectiveness of seven screening strategies for chronic hepatitis B (CHB) patients in China.

**Methods:**

A discrete event simulation model combining a decision tree and Markov structure was developed to simulate a CHB cohort aged ≥40 years on a lifetime horizon and evaluate the costs and health outcomes (quality-adjusted life years [QALYs] gained) of ultrasonography (US), alpha-fetoprotein (AFP), protein induced by vitamin K absence-II (PIVKA-II), AFP+US, AFP+PIVKA-II, GAAD (a diagnostic algorithm based on gender and age combined with results of AFP and PIVKA-II) and GAAD+US. Epidemiologic, clinical performance, utility and cost data were obtained from the literature, expert interviews and real-world data. Uncertainties on key parameters were explored through deterministic and probabilistic sensitivity analyses (DSA and PSA).

**Results:**

Compared with other strategies, GAAD+US detected the most HCC patients at early stage, and GAAD was the screening strategy with the lowest average cost per HCC case diagnosed. Using 3× China's 2022 GDP per capita ($38,233.34) as the threshold, the three strategies of US, GAAD and GAAD+US formed a cost–effectiveness frontier. Screening with US, GAAD, or GAAD+US was associated with costs of $6110.46, $7622.05 and $8636.32, and QALYs of 13.18, 13.48 and 13.52, respectively. The ICER of GAAD over US was $4993.39/QALY and the ICER of GAAD+US over GAAD was $26,691.45/QALY, which was less than 3× GDP per capita. Both DSA and PSA proved the stability of the results.

**Conclusion:**

GAAD+US was the most cost-effective strategy for early HCC diagnosis among CHB patients which could be considered as the liver cancer screening scheme for the high-risk population in China.

Liver cancer is one of the most common malignant tumors worldwide, among which hepatocellular carcinoma (HCC) is the primary pathological type, accounting for 75–85% of liver cancer cases [1]. In 2020, there were 410,000 new cases of liver cancer diagnosed in China, accounting for 45.3% of cases worldwide [2]. According to the Global Burden of Disease (GBD) Study 2017 [3], disability-adjusted life years (DALYs) due to liver cancer reached 11.15 million person-years in China, accounting for 53.7% of global data, seriously threatening the health, and increasing the economic burden, of Chinese patients. Common causes of liver cancer include infection with hepatitis B or C viruses (HBVs/HCVs), non-alcoholic fatty liver disease (NAFLD), alcohol, aflatoxins and cyanobacterial toxins [4]. In mainland China, chronic HBV infection contributes to 84.4% of HCC, and actively promotes hepatocarcinogenesis and HCC progression [5].

In 2018, the 5-year survival rate for patients with HCC was reported to be 14.1% in China [6]. The lack of universal screening for the high-risk population of HCC is the primary reason for the poor long-term survival of HCC patients [7]. When diagnosed, only 33% of patients had early-stage disease (3% at Barcelona Clinic Liver Cancer [BCLC] stage 0 and 30% at BCLC stage A). HCC can be asymptomatic for many years and patients may have advanced disease by the time of diagnosis, resulting in poor prognosis, with treatment generally limited to palliative care [7,8]. In contrast, screening programs for patients with HBV and HCV implemented in Japan facilitated a high proportion (60–65%) of patients with liver cancer being diagnosed at an early stage (i.e., BCLC stage 0 and stage A) [9], with a 5-year survival rate of 50.4% [10]. Therefore, it is essential to launch screening programs for patients with a high risk of developing HCC in China to increase the early diagnosis rate and achieve early treatment, improve prognosis, prolong life expectancy and reduce economic burden due to disease progression. This also aligns with the requirements for promoting early diagnosis and treatment and establishing a long-term screening system that were set out in the Healthy China Action Cancer Control Implementation Plan in 2019 [11].

Recently published Chinese standards for the diagnosis and treatment of primary liver cancer [12] mention various screening strategies including imaging examinations such as abdominal ultrasonography (US), serological tests such as alpha-fetoprotein (AFP) and protein induced by vitamin K absence/antagonist-II (PIVKA-II). The sensitivity of screening with any of these strategies alone is limited (US: 45%; AFP: 63%; PIVKA-II: 64%) [13], especially for early-stage tumors [14]. The use of AFP in addition to US significantly improves the sensitivity of screening and is recommended as the most widely adopted strategy by Chinese guidelines for liver cancer screening but at the cost of reduced specificity [14]; this suggests that many early-stage cases might be missed at diagnosis due to false-negative results, with significant treatment costs generated as a result of disease progression [15]. In this context, new diagnostic strategies and tools for the early detection of HCC represent one of the most promising approaches to reducing the increasing disease burden. PIVKA, which is also mentioned in the Chinese guidelines, can be considered as a supplementary screening strategy [14]. An emerging tool for the detection of HCC includes the biomarker des-γ-carboxy prothrombin (DCP; also known as PIVKA-II) [16], which when combined with AFP, age and gender, forms the GAAD algorithm (Gender, Age, AFP, and DCP [PIVKA-II]). GAAD was developed as a diagnostic algorithm with a higher sensitivity (97%) to estimate the likelihood of HCC in individual patients with chronic liver disease [17]. However, combined strategies may be associated with increased costs. The Chinese guidelines [14] highlight that there is a lack of evidence on health economic evaluations of systemic liver cancer screening strategies and the cost–effectiveness of different screening strategies is still a topic of debate in China. Therefore, this study aims to compare seven screening strategies (US, AFP, PIVKA-II, AFP+US, AFP+PIVKA-II, GAAD and GAAD+US) by developing a health economic model from the Chinese healthcare system perspective to identify the most cost-effective strategy for early detection of liver cancer in patients with CHB in China.

## Methods

### Population

Using a micro-simulation model, we estimated costs and health outcomes for a simulated cohort for HCC screening in China. The simulated cohort was constructed using a targeted population of CHB patients aged 40 years or older in China, who are defined as a high-risk HCC population according to the latest guidelines [12,14], for lifetime simulation.

### Screening strategies

Considering the most recent guidelines and expert consensus recommendations [4,12,14], seven early screening strategies for HCC were compared with each other: US alone, AFP alone, PIVKA-II alone, AFP+US, AFP+PIVKA-II, GAAD and GAAD+US every 6 months in the high-risk HCC population aged 40–70 years.

### Modeling & principal assumptions

**Figure 1.**
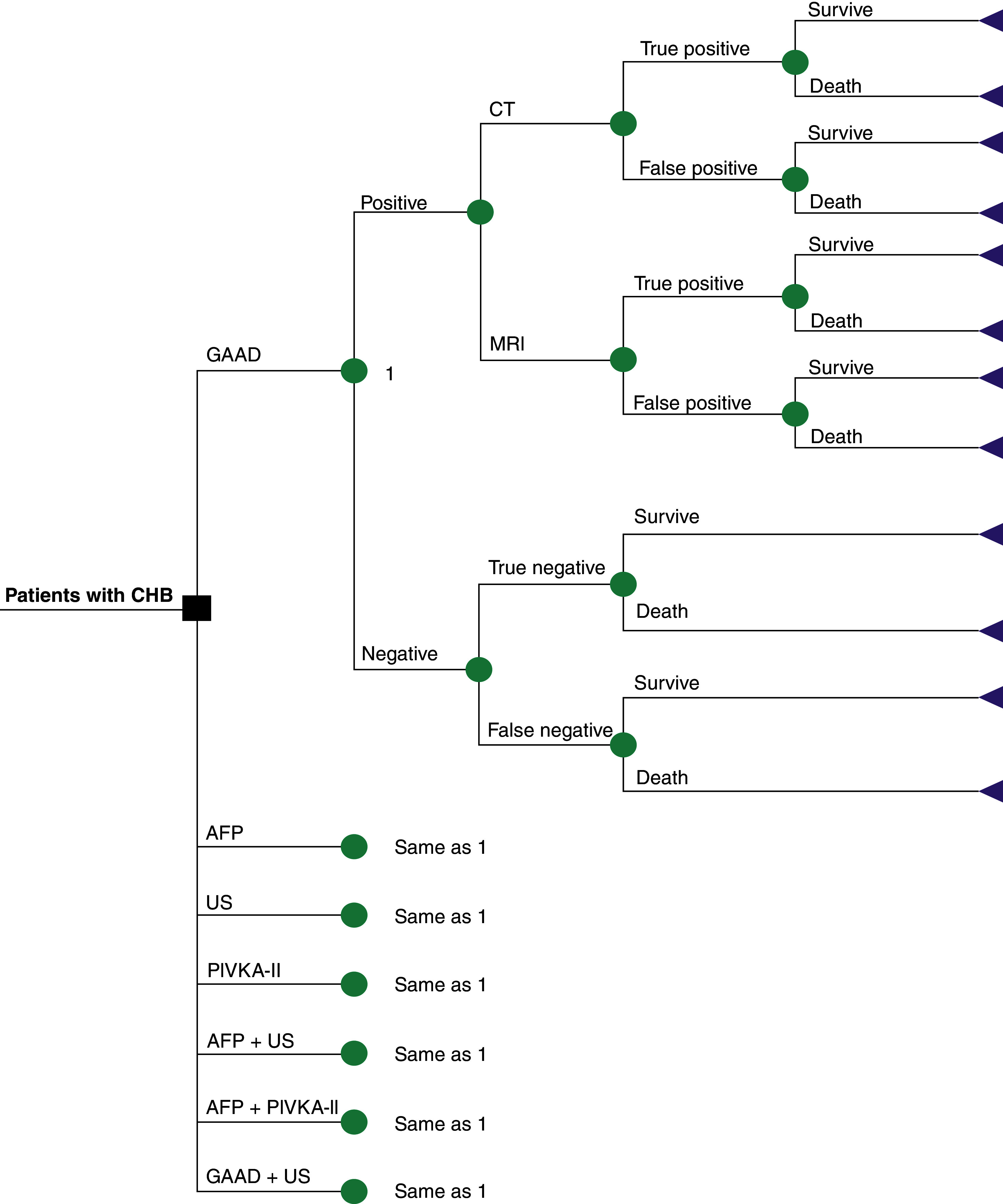
The decision tree model. AFP: Alpha-fetoprotein; CHB: Chronic hepatitis B; GAAD: Gender, Age, AFP and DCP (PIVKA-II); PIVKA-II: Protein induced by vitamin K absence/antagonist-II; US: Ultrasonography.

**Figure 2.**
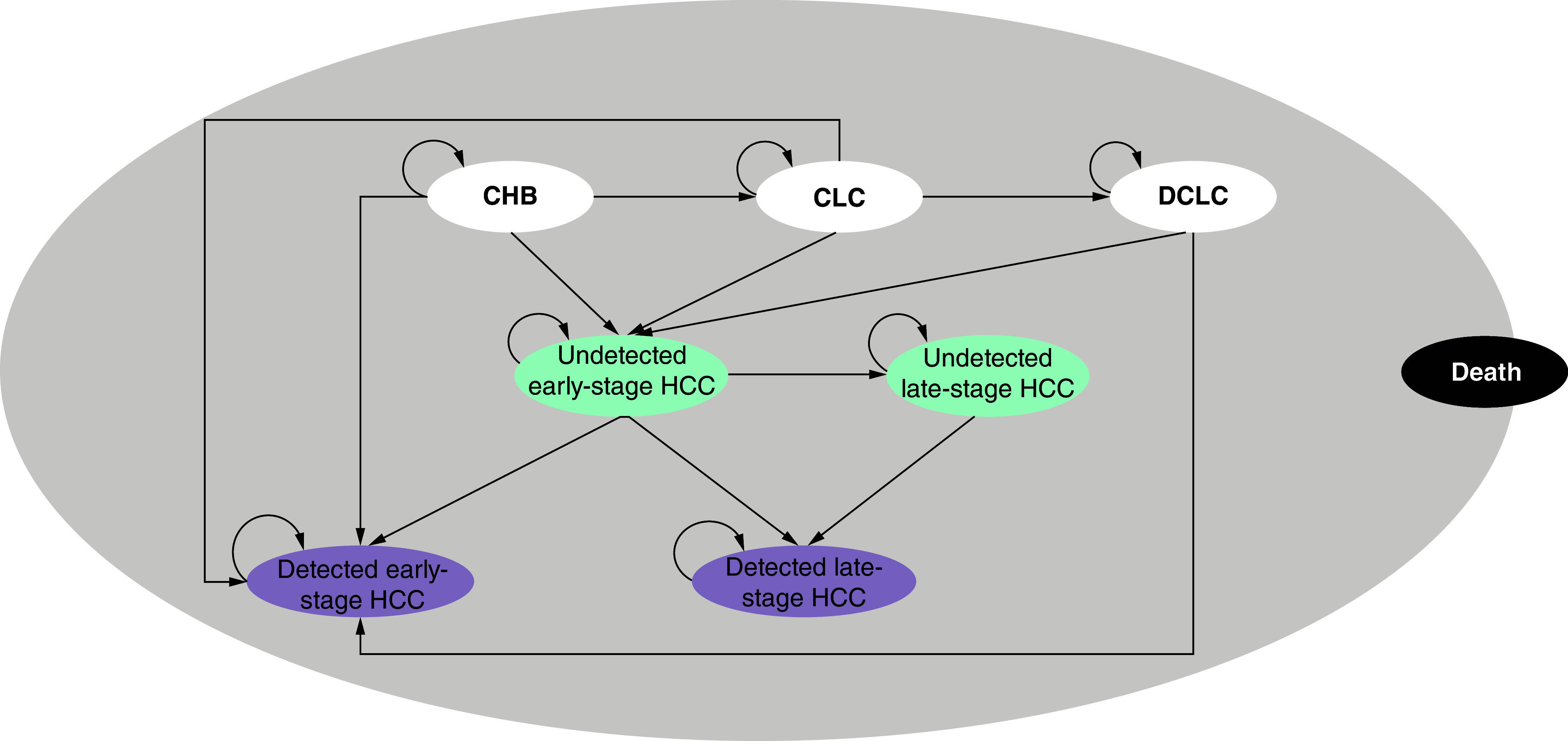
The Markov model. CHB: Chronic hepatitis B; CLC: Compensated liver cirrhosis; DCLC: Decompensated liver cirrhosis; HCC: Hepatocellular carcinoma.

A discrete event simulation (DES) model, which was developed by combining a decision tree and Markov structure (Figures 1 & 2) using Microsoft^®^ Excel 2016, was utilized to assess the cost–effectiveness of the seven screening strategies for liver cancer in the Chinese CHB population from the Chinese healthcare system perspective. The main outcomes were the overall long-term cost of diagnosis and treatment for each strategy, long-term quality-adjusted life year (QALY) and incremental cost–effectiveness ratio (ICER). Costs and health outcomes were discounted at an annual rate of 5% in accordance with the China Guidelines for Pharmacoeconomic Evaluations, version 2020 [18].

The decision-tree model was used to simulate the results of the different screening strategies in CHB patients screened every 6 months with disease progression (Figure 1). The model assumed that there is no difference in adherence to screening strategies, but there are variations in the adherence of patients with different disease statuses to HCC screening. If CHB patients had a positive result on screening, they would receive radiology examinations (i.e., computed tomography [CT] or MRI) with 50% possibility respectively for final diagnosis, which is in accordance with the latest guidelines [12,14]. If the true-positive effect was confirmed by radiology examinations, the patient would receive appropriate treatment and incur the cost, depending on the presence of early- or late-stage HCC. According to expert interviews, it was assumed that, after radiographic confirmation, 5% of patients would still have a false-positive result and undergo surgical treatment and incur the related costs. CHB patients with negative results on screening would continue to be screened for the next cycle.

The Markov model was used to simulate the disease state transition of CHB patients. The BCLC staging system was used to group HCC as early (0/A) and late (B/C/D). Detection can occur in the early or late stages of HCC. The model included eight states: CHB, compensated liver cirrhosis (CLC), decompensated liver cirrhosis (DCLC), undetected early-stage HCC, undetected late-stage HCC, detected early-stage HCC, detected late-stage HCC and death. The model assumed that everyone starts at ‘CHB’ and can only develop to CLC, patients in the state of CLC could progress to DCLC, and patients in the form of CHB, CLC or DCLC could progress to early-stage HCC (undetected or detected). Once the tumor is detected and confirmed by CT or MRI at early-stage HCC, patients could receive one of the following possible treatments: resection, liver transplantation (LT), transarterial chemoembolization (TACE) or radiofrequency ablation (RFA). Patients with undetected early-stage HCC could progress to late-stage HCC (undetected or detected). Following CT- or MRI-confirmed late-stage HCC, patients were considered to be eligible for any of the following therapeutic interventions: resection, LT, TACE, sorafenib or lenvatinib, oxaliplatin-based chemotherapy, or programmed cell death protein-ligand 1 (PD-L1) inhibitors (e.g., atezolizumab plus bevacizumab).

Disease state, adherence to screening and treatment selection for each patient in the simulation cohort were processed based on a DES algorithm. The probability of the event occurring and a random number, generated by Microsoft^®^ Excel 2016, determined whether a related event would occur. If the random number was less than or equal to the probability of the event happening, the event occurs, and *vice versa*. In this model, we assumed the length of a simulation cycle was 6 months; considering the average life expectancy in China (77 years), we ran 120 cycles (i.e., 60-year time horizon) and all patients in the simulation died at the same time.

### Data source

The study was initiated in October 2021 and completed in October 2022. Therefore, the data included in this study were published before October 2022. Model parameters included: diagnostic performance of each screening strategy (Table 1); probability of each state transitions in the Markov model (Table 2); adherence to screening; mortality; screening-related costs (Table 3). All parameters were used to populate the model to generate the cost–effectiveness of the seven screening strategies.

**Table 1.** Sensitivity and specificity of screening strategies for HCC of different stages.

Stage of HCC	Screening strategy	Performance		Stage of HCC	Performance		Data source (year)	Ref.
		Sensitivity	Specificity		Sensitivity	Specificity		
Early-stage HCC	AFP	65.0%	81.0%	Late-stage HCC	63.0%	83.0%	Xing *et al.* (2018)	[19]
	US	45.0%	92.0%		84.0%	92.0%	Tzartzeva *et al.* (2018)	[15]
	PIVKA-II	64.0%	87.0%		68.0%	90.0%	Xing *et al.* (2018)	[19]
	GAAD	78.9%	91.3%		92.9%	91.3%	Chan *et al.* (2021) Roche. (2021)	[20,21]
	AFP+US	63.0%	84.0%		97.0%	84.0%	Tzartzeva *et al.* (2018)	[15]
	AFP+PIVKA	76.1%	90.4%		85.3%	90.4%	Yang *et al.* (2019)	[13]
	GAAD+US	88.5%	88.7%		96.3%	88.7%	Huang *et al.* (2022)	[17]

AFP: Alpha-fetoprotein; GAAD: Gender, Age, AFP and DCP (PIVKA-II); HCC: Hepatocellular carcinoma; PIVKA-II: Protein induced by vitamin K absence/antagonist-II; US: Ultrasonography.

**Table 2.** Annual probability of transition between states in the Markov model.

Probability of transition	Value	Data source (year)	Ref.
CHB to CLC	1.1%	Global Burden of Disease study (2017)	[22]
CHB to early-stage HCC	0.8%	Toy *et al.* (2013)	[23]
CLC to DCLC	3.9%	Toy *et al.* (2013)	[23]
CLC to early-stage HCC	5.0%	Toy *et al.* (2013)	[23]
DCLC to early-stage HCC	7.1%	Toy *et al.* (2013)	[23]
Early-stage HCC to late-stage HCC	5.0%	Mehta *et al.* (2015)	[24]

CHB: Chronic hepatitis B; CLC: Compensated liver cirrhosis; DCLC: Decompensated liver cirrhosis; HCC: Hepatocellular carcinoma.

**Table 3.** Parameters of costs and utility.

Parameters	Value	Data source (year)	Ref.
Cost ($)			
AFP, per test	$6.7	Collection of medical service items and prices of medical institutions in Shanghai (September 2017)	[25]
US, per test	$13.4	Collection of medical service items and prices of medical institutions in Shanghai (September 2017)	[25]
PIVKA-II, per test	$18.6	Collection of medical service items and prices of medical institutions in Shanghai (September 2017)	[25]
GAAD, per test	$25.3	Since the price of GAAD is not currently available in China, the cost is assumed as the sum of the cost of AFP+PIVKA-II, with no additional charge for the algorithm	
CT, per test	$51.0	Collection of medical service items and prices of medical institutions in Shanghai (September 2017)	[25]
Treatment for patients with false-positive results, per capita	$6,311.8	Expert interviews and Zhang *et al.*, 2021	[26]
MRI, per test	$77.8	Collection of medical service items and prices of medical institutions in Shanghai (September 2017)	[25]
CHB treatment, every 6 months per capita	$23.3	Expert interviews and YAOZH.com	[27]
CLC treatment, every 6 months per capita	$155.4	Expert interviews and YAOZH.com	[27]
DCLC treatment, every 6 months per capita	$11,522.3	Expert interviews and YAOZH.com	[27]

Utility			
CHB	0.825	Huang *et al.* (2018)	[28]
CLC	0.761	Huang *et al.* (2018)	[28]
DCLC	0.643	Huang *et al.* (2018)	[28]
Undetected early-stage HCC	0.720	Parikh *et al.* (2020)	[29]
Undetected late-stage HCC	0.675	Parikh *et al.* (2020)	[29]

AFP: Alpha-fetoprotein; CHB: Chronic hepatitis B; CLC: Compensated liver cirrhosis; CT: Computed tomography; DCLC: Decompensated liver cirrhosis; HCC: Hepatocellular carcinoma; PIVKA-II: Protein induced by vitamin K absence/antagonist-II; US: Ultrasonography.

#### Sensitivity & specificity of screening strategy

A complete list of the sensitivity and specificity of each screening strategy, with the respective data sources, is shown in Table 1. If individuals develop HCC under screening, the tumor can be detected or not based on the probability of attendance at regular checkups, the sensitivity and specificity of each screening strategy used, and the probability of symptomatic/incidental detection (e.g., at periodic physical examinations). The probability of symptomatic/incidental detection was 6.2 and 29.3% every 6 months for early- and late-stage HCC, respectively [30].

#### Probability of state transitions

The annual probability of transition between different health states used in the Markov model (Table 2) was derived from published health economics and epidemiological studies [21–23], converted into transition probability every 6 months, and applied to each simulation cycle. The conversion calculation of transition probability is shown in Supplementary Materials.

#### Adherence

Data for adherence to HCC screening were obtained from the published literature and expert interviews. After completing the target literature review, we found that there was no published literature reported on variations in adherence rates of Chinese CHB patients among the included seven screening programs. According to a published meta-analysis [31] and expert interviews, patients with CHB had a lower adherence rate than patients with cirrhosis, so we assumed that there is no difference between screening strategies but there are differences between health states. The adherence of Chinese patients with CHB in HCC screening was 58% [32], a coefficient of 1.34 was used to adjust [33] the adherence of Chinese patients with CLC and DCLC.

#### Mortality

Because the mortality rate of CHB is quite low, it was assumed that mortality in patients with CHB was equal to all-cause mortality in the general population. The annual mortality rate in patients with CLC and DCLC sourced from the published literature was 3.5% [34] and 9.9% [35], respectively. The mortality of patients with HCC was generated from Kaplan-Meier survival curves for overall survival in clinical trials of different treatments fitted and extrapolated by R Studio software using seven common distributions (exponential, Weibull, Gompertz, log-logistic, log-normal, gamma and generalized-gamma) with the selected function of best fit. The optimal parameter model was selected through goodness-of-fit tests and plausibility checks. The survival of early-stage HCC patients follows the Log-normal distribution, whereas the survival of late-stage HCC patients follows the Gompertz distribution.

#### Cost & utility

Since the model was constructed from the healthcare system perspective, only direct medical costs, including costs of screening and treatment, were considered in this study. Costs of screening were defined as the cost of each screening strategy, with representative data sourced from the collection of medical service items and prices of medical institutions in Shanghai used as the parameters for the base-case analysis. The probabilities of HCC patients confirmed by radiology examination, including patients with a false-positive result, receiving different treatment strategies, and the costs of each treatment strategy, were acquired from expert interviews. Treatment cost for patients with false-positive results was defined as the cost of surgical treatment [26]. The costs of treatment for patients in the states of CHB, CLC and DCLC, including all direct medical costs incurred during outpatient and inpatient stays, are presented in Table 3. All costs are presented in $, with an average exchange rate of $1 = 6.7261 Chinese Yuan in 2022. The utility values for each health state were sourced from published health economics studies (Table 3). The utility values of patients with detected early- and late-stage HCC were based on the treatment they received (some of these data were based on assumptions, not shown in Table 3). Costs and utility values were discounted at an annual rate of 5%.

### Sensitivity analysis

The impact of parameter uncertainty on results was explored with one-way sensitivity analyses and resumed in Tornado plots. The ranges of variations were determined to be between the upper and lower limits of the 95% confidence intervals reported in the literature sources for the probability of transition between different health states, sensitivity and specificity of each screening strategy, and the utility of patients with CHB, CLC or DCLC (Supplementary Table 1). Regarding testing costs for AFP, PIVKA-II, US, CT and MRI, the highest and the lowest medical service prices in Shanghai, Guangzhou and Zhejiang were taken as the upper and lower limits, respectively. For other parameters, the ranges of variations were set to ±20%. Probabilistic sensitivity analysis was performed to analyze joint parameter uncertainty and to build cost–effectiveness acceptability curves (CEACs). We assumed a normal distribution for the upper and lower limits of age in patients requiring HCC screening, a gamma distribution for the parameters of costs and a beta distribution for other parameters and 1000-times Monte Carlo simulations were performed for PSA.

## Results

### Screening results & costs of different screening strategies

Since the DES model requires the use of random numbers generated by Excel, the results for each model calculation are random. The model results were tested to be more robust under 5000 simulations, so the average of 5000 simulations was used for the report to ensure stability.

After lifetime simulations of 5000 patients in the model, 1724 patients progressed to HCC. The number of patients with detected HCC, the proportion of detected early-stage HCC patients, average cost per HCC case diagnosed and treatment costs due to false-positive results for each strategy are presented in Table 4. GAAD+US showed the highest rate of early detection with 64.9% of HCC cases at early-stage, followed by GAAD (62.2%), AFP+PIVKA-II (61.4%), AFP (57.2%), PIVKA-II (57.0%), AFP+US (56.7%) and US (48.3%). GAAD was the screening strategy with the lowest average cost per HCC case diagnosed, followed by AFP+PIVKA-II and GAAD+US. The lowest cost of treatment for false-positive HCC was associated with US, followed by GAAD.

**Table 4.** Screening efficiency and cost for false-positive results for each strategy.

No.	Screening strategy	Patients with detected HCC (n)	Proportion of early-stage HCC	Average cost per HCC case diagnosed ($)	Treatment cost due to false-positive results^†^ ($)
1	US	832	48.3%	17.09	4,998,474.3
2	PIVKA-II	982	57.0%	16.94	8,116,800.8
3	AFP+US	977	56.7%	17.40	10,032,045.9
4	AFP	986	57.2%	17.32	11,962,840.5
5	AFP+PIVKA-II	1059	61.4%	16.50	6,015,406.9
6	GAAD	1073	62.2%	16.32	5,440,966.6
7	GAAD+US	1118	64.9%	16.91	7,061,661.1

† Not discounted.

AFP: Alpha-fetoprotein; GAAD: Gender, Age, AFP and DCP (PIVKA-II); HCC: Hepatocellular carcinoma; PIVKA-II: Protein induced by vitamin K absence/antagonist-II; US: Ultrasonography.

### Base-case results

The base-case results are presented in Table 5. GAAD+US was the strategy with the most QALYs per person, followed by GAAD, AFP+PIVKA-II, AFP, PIVKA-II, AFP+US and US. In terms of costs, GAAD+US was the costliest screening strategy, followed by GAAD, AFP+PIVKA-II, AFP, AFP+US, PIVKA-II and US. The ICER threshold was defined as three-times the gross domestic product (GDP) per capita in China ($12,714.11 in 2022), which was $38,233.34/QALY.

**Table 5.** Basic analysis results.

No.	Screening strategy	Cost ($)	QALYs	Comparison	ICER ($ per QALY gained)
1	US	6110.46	13.177		
2	PIVKA-II	7277.37	13.369	2 vs 1	6090.82
3	AFP+US	7461.31	13.354	–	-
4	AFP	7523.02	13.374	4 vs 2	43,209.27
5	AFP+PIVKA-II	7594.19	13.463	5 vs 2	3351.72
				5 vs 1	5185.89
6	GAAD	7622.05	13.480	6 vs 5	1677.25
				6 vs 1	4993.39
7	GAAD+US	8636.32	13.518	7 vs 6	26,691.45

AFP: Alpha-fetoprotein; GAAD: Gender, Age, AFP, and DCP (PIVKA-II); ICER: Incremental cost–effectiveness ratio; PIVKA-II: Protein induced by vitamin K absence/antagonist-II; QALY: Quality-adjusted life year; US: Ultrasonography.

The seven screening strategies were ranked in descending order of cost per capita. AFP+US (no. 3) was strictly dominated by PIVKA-II (No. 2) due to a higher price and lower effectiveness and, therefore, was excluded from the analysis. Among the remaining six strategies, PIVKA-II (no. 2) was cost effective compared with US (No. 1), with 0.19 QALY gained at an incremental cost of $1166.91, resulting in an ICER of $6090.82/QALY gained, which was lower than three-times GDP per capita. Compared with PIVKA-II (no. 2), AFP (no. 4) was not cost effective and was excluded, with 0.01 QALY gained at an incremental cost of $245.65, resulting in an ICER of $43,209.27/QALY gained, which was higher than three-times GDP per capita. Compared with PIVKA-II (no. 2), AFP+PIVKA-II (no. 5) was cost effective, with 0.10 QALY gained at an incremental cost of $316.82, resulting in an ICER of $3351.72/QALY, which is lower than three-times GDP per capita. Compared with AFP+PIVKA-II (no. 5), GAAD (no. 6) was cost effective, with 0.02 QALY gained at an incremental cost of $27.86, resulting in an ICER of $1677.25/QALY, which was lower than three-times GDP per capita. Compared with US (no. 1), the ICER of GAAD (No. 6) was $4993.39/QALY, which was lower than PIVKA-II (no. 2; $6090.82/QALY) and AFP+PIVKA-II (no. 5; $5185.89/QALY). Therefore, PIVKA-II (no. 2) and AFP+PIVKA-II (no. 5) were excluded and defined as extended-dominated strategies. Compared with GAAD (no. 6), GAAD+US (no. 7) was cost effective, with 0.04 QALY gained at an incremental cost of $1014.27, resulting in an ICER of $26,691.45/QALY, which was less than three-times GDP per capita.

**Figure 3.**
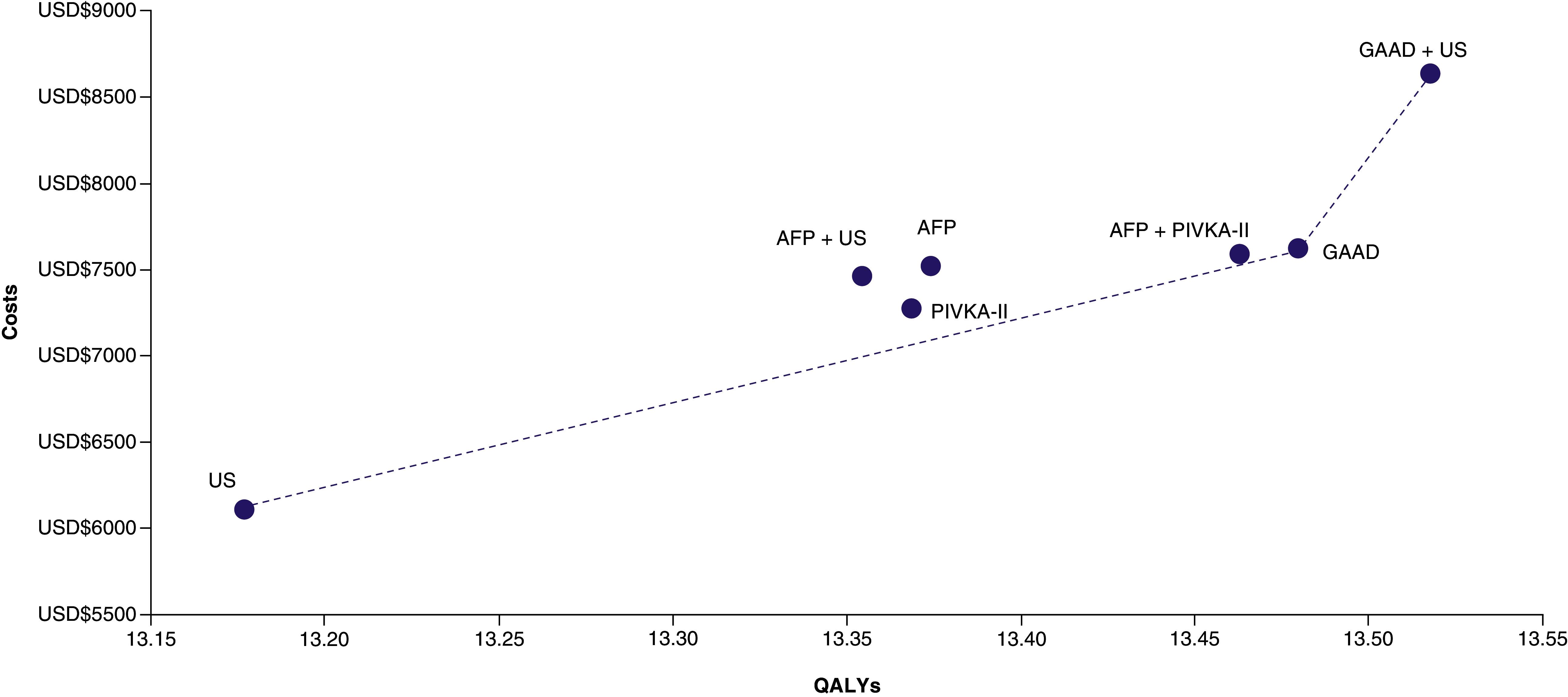
Cost–effectiveness frontier. AFP: Alpha-fetoprotein; GAAD: Gender, Age, AFP and DCP (PIVKA-II); PIVKA-II: Protein induced by vitamin K absence/antagonist-II; QALY: Quality-adjusted life year; US: Ultrasonography.

US, GAAD, and GAAD+US comprised the cost–effectiveness frontier (Figure 3), whereas GAAD+US was the most cost-effective screening strategy for HCC in patients with CHB in China.

### Sensitivity analysis

#### One-way sensitivity analysis

**Figure 4.**
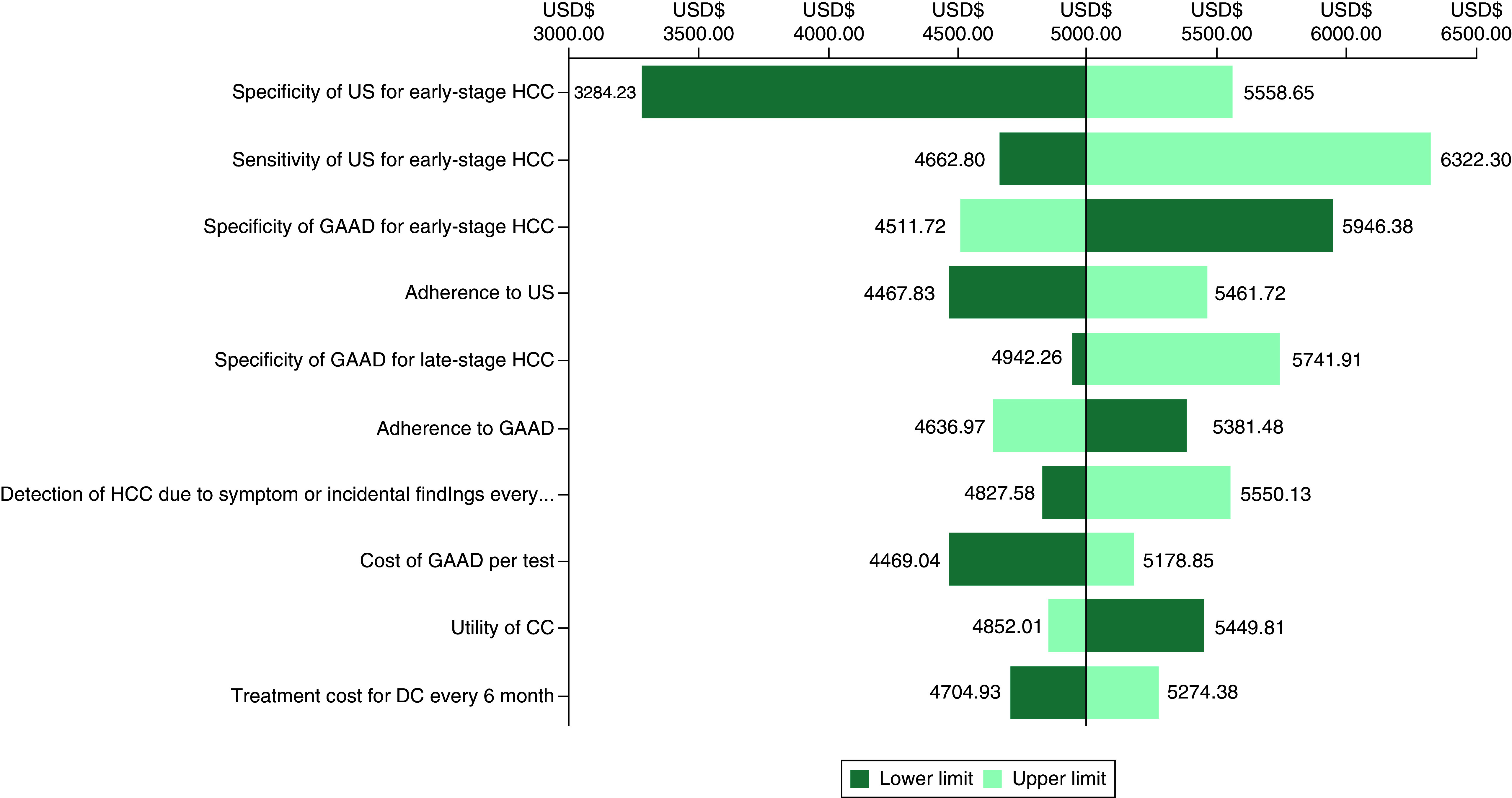
Tornado diagram of one-way sensitivity analysis (GAAD vs US). CLC: Compensated liver cirrhosis; DCLC: Decompensated liver cirrhosis; GAAD: Gender, age, AFP and DCP (PIVKA-II); HCC: Hepatocellular carcinoma; ICER: Incremental cost–effectiveness ratio; US: Ultrasonography.

Considering the results of the cost–effectiveness analysis, namely US, GAAD and GAAD+US forming a cost–effectiveness frontier, we conducted a one-way sensitivity analysis for the cost–effectiveness comparisons of GAAD (no. 6) versus US (no. 1) and GAAD+US (no. 7) versus GAAD (no. 6) (Figure 4 & Supplementary Figure 1).

When GAAD was compared with US, the ICER was lower than three-times GDP per capita, regardless of parameter variations within the prespecified range. A tornado diagram of one-way sensitivity analysis for the ten most influential parameters is presented in Figure 4. Generally, the most influential variables are the specificity and sensitivity of US and the specificity of GAAD for early-stage HCC. The robustness of the results was confirmed.

#### Probabilistic sensitivity analysis

**Figure 5.**
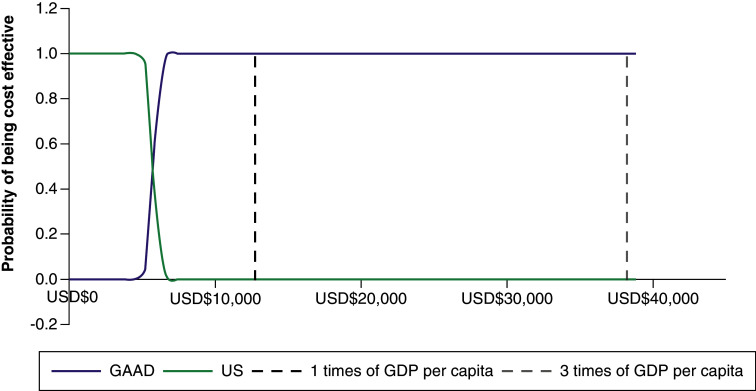
Probability of being the most cost-effective option for different willingness to pay thresholds for GAAD versus US. GAAD: Gender, Age, Alpha-fetoprotein and DCP (protein induced by vitamin K absence/antagonist-II); GDP: Gross domestic product; US: Ultrasonography.

**Figure 6.**
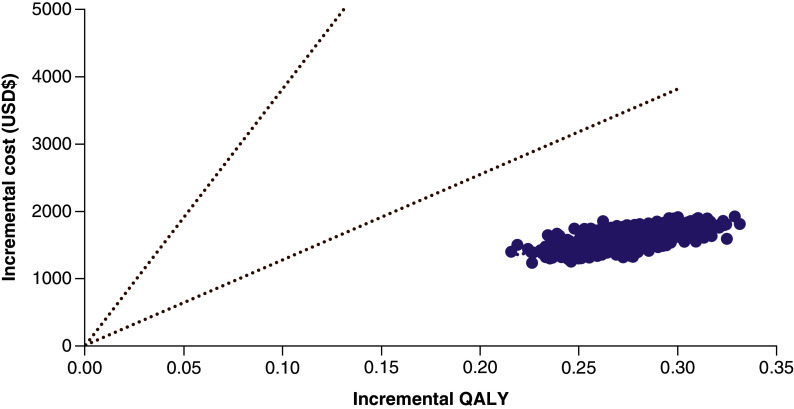
Incremental cost–effectiveness plane of GAAD versus US. GAAD: Gender, Age, AFP and DCP (PIVKA-II); GDP: Gross domestic product; QALY: Quality-adjusted life year; US: Ultrasonography.

We also conducted a probabilistic sensitivity analysis for the two comparisons mentioned above (Figures 5 & 6 & Supplementary Figures 2 & 3). CEACs were yielded based on 1000 Monte Carlo simulations. When a willingness-to-pay (WTP) threshold was equal to three-times GDP per capita ($38,233.34), the probability of achieving cost–effectiveness for GAAD compared with US was 99.9% (Figure 5). Figure 6 contains the comparisons between GAAD and US made in a cost–effectiveness plane, where the x-axis shows the gains in QALYs per individual and the y-axis the additional costs per individual. It showed that 99.9% of results were in the northeast quadrant for GAAD versus US, meaning that GAAD continued to dominate US in each simulation and was 99.9% cost-effective at all WTP thresholds.

## Discussion

Our approach was to build a simulation model, looking to include all relevant considerations to estimate patient health outcomes and cost consequences for different HCC screening strategies for CHB patients in China: US, AFP, PIVKA-II, AFP+US, AFP+PIVKA-II, GAAD and GAAD+US. Our results suggest that HCC screening would be cost-effective with the GAAD algorithm, or when accompanying US with GAAD, but it would not be the case with US or AFP+US. These results are especially relevant when compared with previous findings as we used the diagnostic algorithm GAAD and GAAD+US; screening strategies with higher sensitivity can effectively reduce the amount of underdiagnosis and false-negative results in high-risk patients, allowing more patients to be detected at an early stage of HCC, thus increasing the chance of patients receiving curative treatment to improve prognosis and survival, avoiding the heavy economic burden arising from disease progression to advanced stages. On the other hand, with higher specificity, the diagnostic algorithm could save unnecessary medical costs associated with false-positive diagnoses [36]. Moreover, the diagnostic algorithm can improve patients' overall quality of life, with more patients being diagnosed and treated early to reduce the social and economic burden of HCC and, thus, improving social labor productivity.

Health economics studies focusing on HCC screening in patients with CHB and comparing different screening protocols have been published in China and other countries [14]. However, the perspectives, comparators and screening strategies varied among studies, confusing the decision-making process. Existing studies in China and overseas have focused on AFP combined with US as a screening strategy. For example, a community-based survey conducted in Shanghai demonstrated that the cost of screening with AFP+US screening was $89,132 per HCC case detected [37]. In another HCC screening study conducted in Shanghai in patients aged 35 to 59 years with CHB or who were hepatitis B virus surface antigen-positive, the ICER for one AFP + US screening every 6 months compared with no screening was $214 which was lower than per capita GDP in China during the study period (1993–1997) and was considered cost-effective [38]. In a study conducted in Taiwan, screening for HCC in the whole population and applying biochemistry testing plus AFP+US in a high-risk group (including patients with CHB) was a more cost-effective regimen than no screening [39]. Moreover, Garay *et al.* reported that, compared with AFP+US, screening for HCC using GAAD in patients with CLC was a strictly dominant strategy in the United Kingdom, with lower cost and higher efficacy [40].

The current study has several limitations. First, the model simplifies clinical practice by several vital assumptions (e.g., 100% adherence to treatment in patients with CHB, CLC, and DCLC). Second, due to the need for more utility data for Chinese patients in some health states, the model was calculated by assumptions or using data from other countries, which may differ from the actual situation among the Chinese population. Third, the model's screening and treatment costs originate from publicly available medical service prices in Shanghai and expert interviews, which may not represent the nationwide situation. Finally, the diagnostic performance parameters were mainly derived from individual studies, representing the highest quality data available. However, the parameters and assumptions of the model were validated by one-way and probabilistic sensitivity analysis, and the results were found to be robust.

Given the high prevalence of liver disease in China, with more than 300 million people estimated to have potential chronic liver disease, it is difficult and costly to implement large-scale population-wide screening. Establishing screening paradigms in high-risk groups may be effective and efficient; the multidimensional evidence on the effectiveness, safety, accessibility and affordability of screening strategies should be considered [14]. This will not only facilitate the implementation and promotion of an early screening system for liver cancer but also play an essential role in promoting the prevention and treatment of liver disease, tackling liver cancer and improving public health.

## Conclusion

It is indeed important to develop cost-effective screening strategies that are tailored to China's national conditions. The findings from this study suggest that, from a cost-effective perspective, GAAD+US would be the preferred liver cancer screening scheme for the high-risk population in China. This will not only promote the implementation and adoption of early liver cancer screening mechanisms, but it will also play a crucial role in promoting liver disease prevention and treatment. Achieving the goal of ‘eliminating hepatitis’ and improving public health would have tremendous significance for the Chinese population.

## Summary points

- Regular screening is proposed as an essential method to improve the early diagnosis of and prognosis of patients with hepatocellular carcinoma (HCC). Several screening strategies are used clinically, among which a single system alone demonstrates limited performance, while combined screening strategies incur higher costs.
- This study assessed the cost–effectiveness of seven different screening strategies for patients with chronic hepatitis B (CHB) in China: ultrasonography (US), alpha-fetoprotein (AFP), protein induced by vitamin K absence-II (PIVKA-II), AFP+US, AFP+PIVKA-II, GAAD and GAAD+US.
- A discrete event simulation model combining a decision tree and Markov structure was developed to simulate a CHB cohort aged ≥40 years on a lifetime horizon from the Chinese healthcare system perspective.
- GAAD+US detected the most HCC patients and the highest proportion of early HCC patients compared with other strategies.
- Using a threshold of 3× China's 2021 GDP per capita ($38,233.34), US, GAAD and GAAD+US formed a cost–effectiveness frontier. Screening with US, GAAD, or GAAD+US was associated with costs of $6110.46, $7622.05, and $8636.32, respectively and quality-adjusted life-years (QALYs) of 13.18, 13.48 and 13.52, respectively. The model results were stable to sensitivity analyses.
- Among the seven diagnostic strategies evaluated in China, GAAD+US was the most cost-effective strategy for early diagnosis of HCC.

## Supplementary Material






