# Supplementary material for: Early-stage hepatocellular carcinoma screening in patients with chronic hepatitis B in China: a cost–effectiveness analysis
Source: J Comp Eff Res. 2024 Feb 28;13(4):e230146. doi: 10.57264/cer-2023-0146 (PMC11044951; doi:10.57264/cer-2023-0146)
Supplement: Supplementary file 1 [file cer-13-230146-s1.pdf]

Presented at ISHVLD GHS 2021, 18–20 June, 2021. Global Hepatitis Summit.

Performance Evaluation of the Elecsys® GAAD Assay for the Detection of Hepatocellular Carcinoma Across Different Disease Stages and Etiologies

Henry LY Chan,[1] Arndt Vogel,[2] Thomas Berg,[3] Enrico N De Toni,[4] Masatoshi Kudo,[5] Jörg Trojan,[6] Anja Eiblmaier,[7] Hanns-Georg Klein,[8] Johannes Kolja Hegel,[9] Konstantin Kroeniger,[10] Kairat Madin,[10] Ashish Sharma[11], Teerha Piratvisuth[12]

[1]The Chinese University of Hong Kong, Hong Kong, China; [2]Medizinische Hochschule Hannover, Hannover, Germany; [3]Universitätsklinikum Leipzig, Leipzig, Germany; [4]Department of Medicine II, University Hospital, Ludwig Maximilian University of Munich, Munich, Germany; [5]Kindai University, Osaka, Japan; [6]Goethe Universitat Frankfurt, Frankfurt, Germany; [7]Microcoat Biotechnologie GmbH, Bernried, Germany; [8]Zentrum fur Humangenetik und Laboratoriumsdiagnostik, Munich, Germany; [9]Labor Berlin Charité Vivantes Services GmbH, Berlin, Germany; [10]Roche Diagnostics GmbH, Penzberg, Germany; [11]Roche Diagnostics International AG, Rotkreuz, Switzerland; [12]Prince of Songkla University, Hat Yai, Thailand

Introduction

- Hepatocellular carcinoma (HCC) is a leading cause of cancer-related mortality (~830,000 deaths/year)[1,2] and typically develops in patients with chronic liver diseases (CLDs)[3]
  - Cirrhosis, which can be caused by chronic viral infection (hepatitis B [HBV] or C [HCV]), alcoholic or non-alcoholic steatohepatitis (NASH), accounts for 80–90% of cases regardless of etiology[4]
- Current guidelines recommend routine surveillance of high-risk patients every 6 months using ultrasonography; however, effectiveness for the detection of early-stage HCC is limited[5]
- Protein-induced by vitamin K absence-II (PIVKA-II) and alpha-fetoprotein (AFP) have been identified as serum biomarkers linked to HCC, but these biomarkers have been inconsistently incorporated into guidelines[5–7]
- The Elecsys® **GAAD** algorithm (Roche Diagnostics International Ltd, Rotkreuz, Switzerland) is an *in vitro* diagnostic multivariate index assay combining the following to provide a semi-quantitative result:

**G**ender	**A**ge	**A**FP*	**D**CP (PIVKA-II)*

**quantitative measurements (using respective Elecsys assays) in human serum and plasma*

Objective

To assess the clinical performance of the Elecsys GAAD algorithm to differentiate HCC and benign CLD, according to disease stage and etiology

Methods

- Patients and disease controls aged ≥18 years were prospectively enrolled at seven clinics in the People's Republic of China, Germany, Japan and Thailand.
- Eligible HCC cases had first-time HCC diagnosis confirmed radiologically according to national guidelines, or by liver biopsy. Key exclusion criteria were: presence of any other cancer, except non-melanoma skin cancer (NMSC); recurrent HCC; current or previous treatment for HCC. The key exclusion criterion in disease controls was presence of any cancer except NMSC
  - HCC cases were also grouped according to Barcelona Clinic Liver Cancer (BCLC) staging (early, stages 0/A; late, stages B/C/D).
- Eligible disease controls had absence of HCC confirmed by imaging within 12 months prior, and presence of: cirrhosis; non-cirrhotic chronic HBV infection; non-cirrhotic chronic HCV infection; non-cirrhotic NASH.
- Serum samples were collected ≥1 day prior to surgery and the measurements of Elecsys PIVKA-II (ng/mL) and Elecsys AFP (ng/mL) assays were run on cobas e 601 analyzer in three experimental runs at Microcoat GmbH (Bernried, Germany).
- The clinical performance of the Elecsys GAAD algorithm was compared with that of the Elecsys AFP assay alone. Cut offs for the detection of HCC were:
  - Elecsys GAAD score of **2.57** (generated score between 0–10)
  - Elecsys AFP concentration of **20 ng/mL**
- All clinical information was collected in an electronic data capture system and informed consent provided.
- Performance was assessed using receiver operating characteristic (ROC) analysis and AUC values were calculated.
- Sensitivity and specificity at the established cut-offs were determined and derived 95% confidence intervals (CIs) computed from the binomial distribution using the Clopper-Pearson method[8]
  - One-sided McNemar test was used for comparison of sensitivities

Results

Participants

- A total of 470 patients were screened; of these, 156 HCC cases and 208 disease controls were enrolled in the study (**Table 1**).
- In the HCC cohort, mean age was 62.6 years, 130 (83.3%) were male and 130 (83.3%) had cirrhotic etiology.
  - Seventy-one (45.5%) had early-stage and 85 (54.5%) had late-stage HCC.
- In the control cohort, mean age was 52.2 years, 126 (60.6%) were male and 79 (38.0%) had cirrhotic etiology.

Table 1. Participant demographics and clinical characteristics

Characteristics	Control (N=208)	HCC (N=156)
**Mean age, years (SD)**	52.2 (12.3)	62.6 (9.9)
**Gender, n (%)**		
Male	126 (60.6)	130 (83.3)
**Race, n (%)**		
Asian	99 (47.6)	71 (45.5)
White	101 (48.6)	84 (53.8)
Black or African American	3 (1.4)	0
Other	0	0
Missing	5 (2.4)	1 (0.6)
**Disease etiology, n (%)**		
Cirrhosis	79 (38.0)	130 (83.3)
HBV	72 (34.6)	11 (7.1)
HCV	27 (13.0)	3 (1.9)
NASH	30 (14.4)	1 (0.6)
ALD	0	2 (1.3)
Other	0	9 (5.8)
**BCLC stage, n (%)**		
0	–	10 (6.4)
A	–	61 (39.1)
B	–	26 (16.7)
C	–	51 (32.7)
D	–	8 (5.1)

ALD, non-cirrhotic alcoholic liver disease; BCLC, Barcelona Clinic Liver Cancer; HBV, hepatitis B virus; HCC, hepatocellular carcinoma; HCV, hepatitis C virus; NASH, non-alcoholic steatohepatitis; SD, standard deviation.

Clinical Performance

- Distribution of the Elecsys GAAD score results by cases and controls **(Figure 1A)**, BCLC staging **(Figure 1B)**, etiology **(Figure 1C)** and geographical region **(Figure 1D)** are shown below.
  - The Elecsys GAAD score results were effective at distinguishing between HCC cases and CLD controls, and correlated with HCC disease stage.

Figure 1. Distribution of Elecsys GAAD score by cases and controls (A), BCLC staging (B), etiology (C) and geographical region (D).

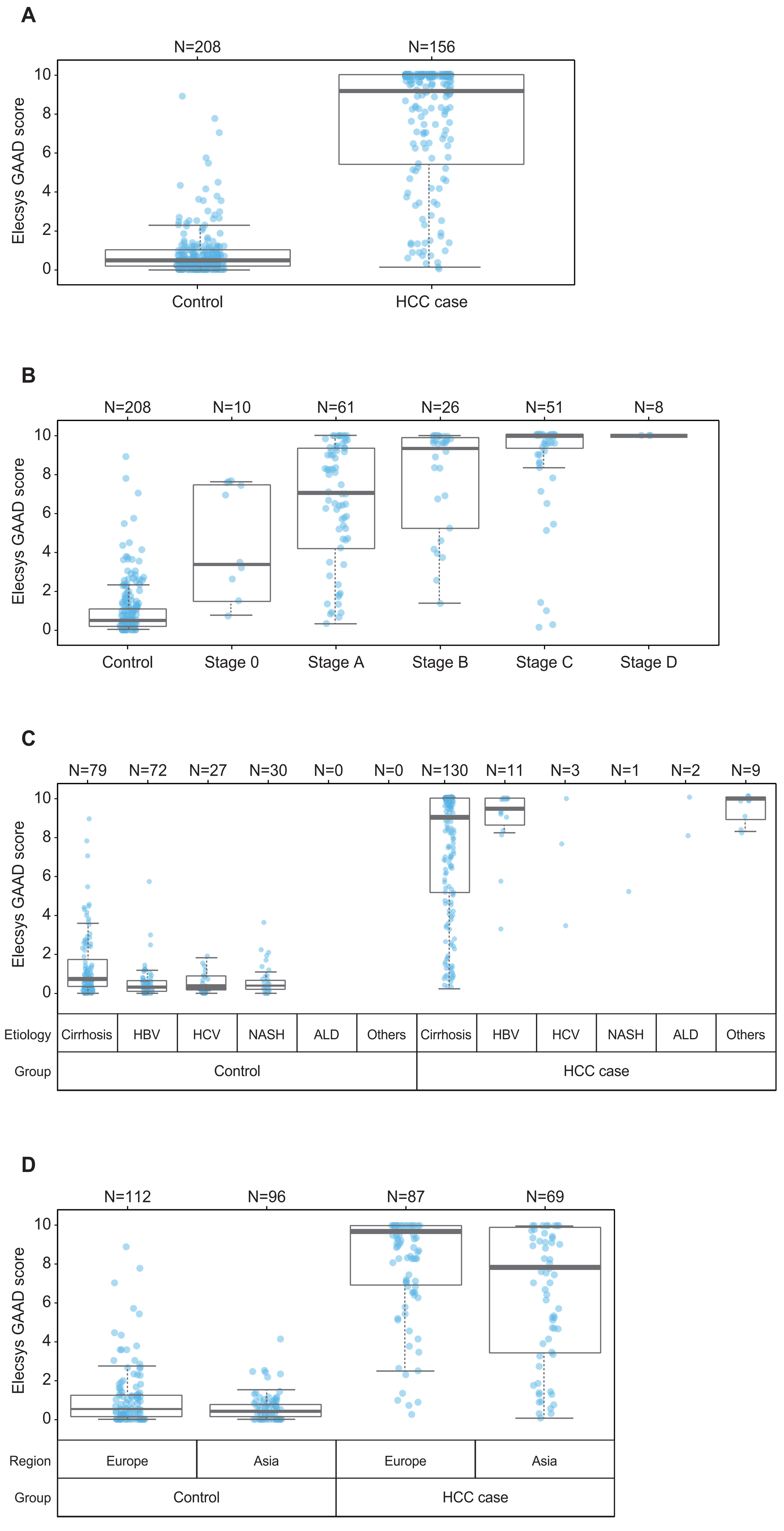

AFP, alpha-fetoprotein; ALD, non-cirrhotic alcoholic liver disease; BCLC, Barcelona Clinic Liver Cancer; HBV, hepatitis B virus; HCC, hepatocellular carcinoma; HCV, hepatitis C virus; NASH, non-alcoholic steatohepatitis.

- The Elecsys GAAD score showed better clinical performance for detecting both early- and all-stage HCC versus Elecsys AFP alone **(Figure 2)**.

Figure 2. ROC plot of Elecsys AFP assay and Elecsys GAAD score for discriminating between disease control and (A) early-stage or (B) all-stage HCC patients

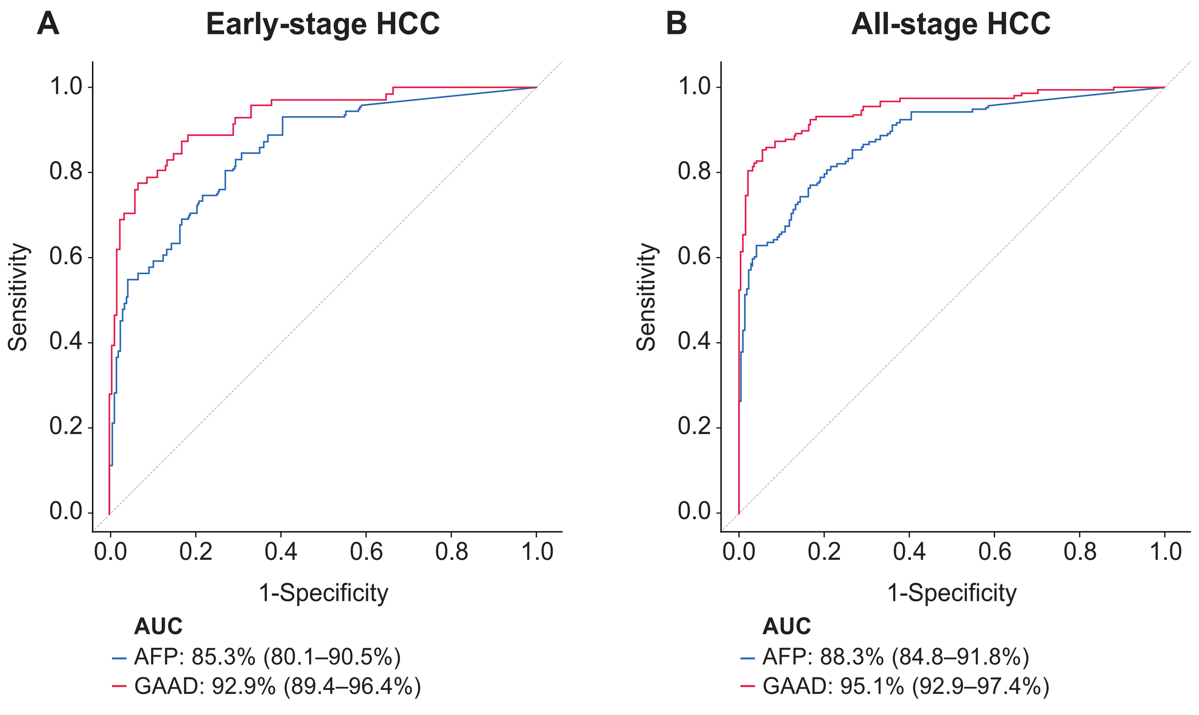

AFP, alpha-fetoprotein; HCC, hepatocellular carcinoma; ROC, receiver operating characteristic.

- The sensitivity for detecting early- and all-stage HCC, respectively, was 41 and 34 percentage points higher using the Elecsys GAAD algorithm compared with Elecsys AFP assay alone at a high specificity (>90%) **(Table 2)**.

Table 2. Clinical performance of Elecsys AFP assay and Elecsys GAAD algorithm for the detection of early- and all-stage HCC.

	Early-stage HCC (N=71)		All-stage HCC (N=156)	
	Sensitivity % (95% CI)	Specificity % (95% CI)	Sensitivity % (95% CI)	Specificity % (95% CI)
**Elecsys AFP assay (cut off: 20 ng/mL)**	38.0 (26.8–50.3)	98.1 (95.2–99.5)	52.6 (44.4–60.6)	98.1 (95.2–99.5)
**Elecsys GAAD score (cut off: 2.57)**	78.9 (67.6–87.7)	91.4 (86.7–94.8)	86.5 (80.2–91.5)	91.4 (86.7–94.8)

AFP, alpha-fetoprotein; HCC, hepatocellular carcinoma.

- The Elecsys GAAD algorithm was effective at distinguishing early- and all-stage HCC, regardless of etiology or geographical region **(Figure 3)**.

Figure 3. ROC plot of Elecsys GAAD score for discriminating between HCC patients and disease controls, by etiology group (A and B) and by region (C and D)

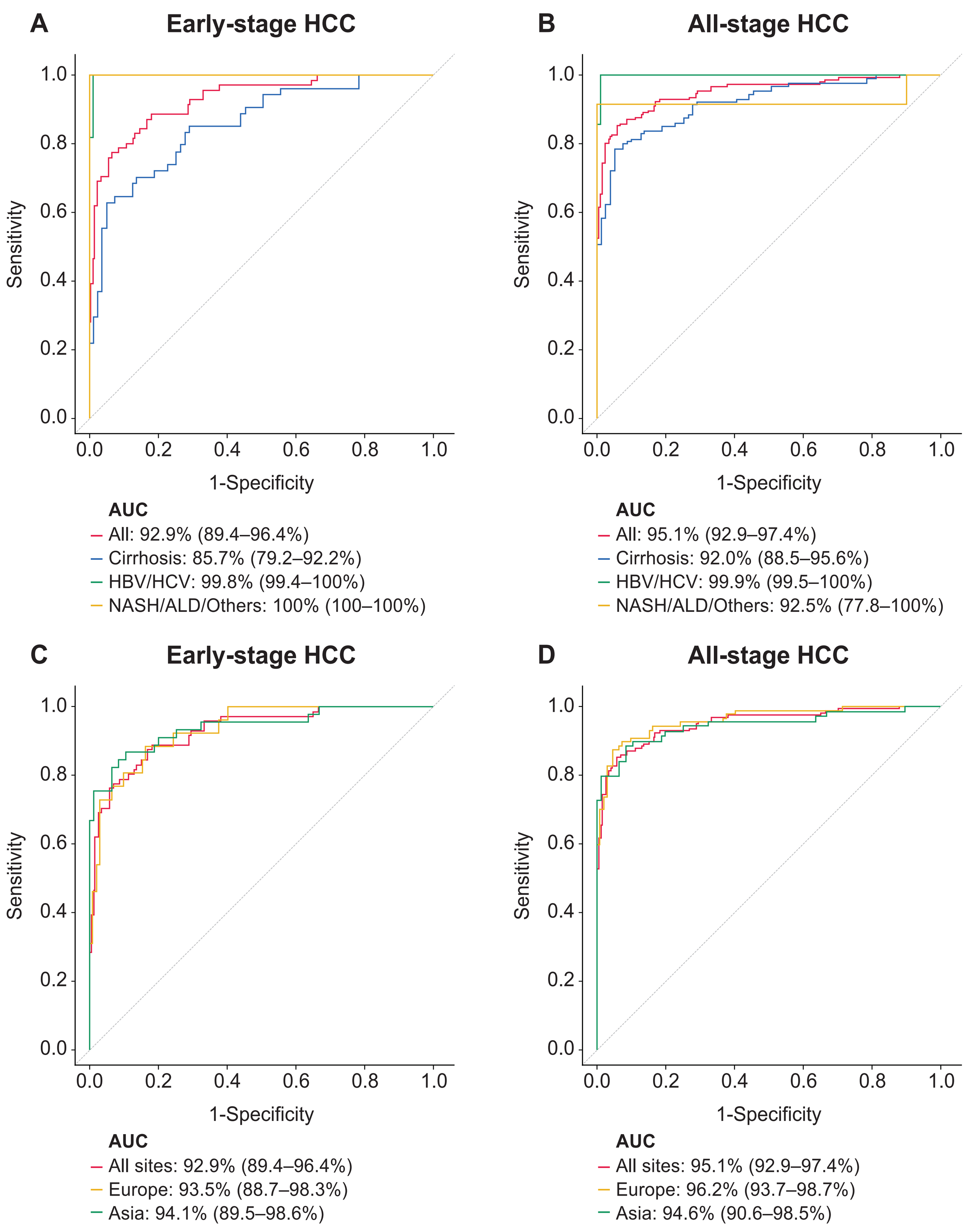

ALD, non-cirrhotic alcoholic liver disease; AUC, area under the curve; HBV, hepatitis B virus; HCC, hepatocellular carcinoma; HCV, hepatitis C virus; NASH, non-alcoholic steatohepatitis; ROC, receiver operating characteristic.

Conclusions

- **The Elecsys GAAD algorithm, combining PIVKA-II and AFP, plus age and gender, demonstrated good clinical performance in differentiating HCC and benign CLD, across all disease stages and etiologies**
- **For the detection of both early- and all-stage HCC, the Elecsys GAAD score performed better than Elecsys AFP assay alone**
- **These findings provide supporting evidence for the use of the Elecsys GAAD score as an aid in the detection of early-stage HCC in patients with CLD undergoing surveillance**

References

1. Davis GL et al. *Proc (Bayl Univ Med Cent)* 2008;21(3):266–280
2. WHO. Cancer. Available from: https://www.who.int/news-room/fact-sheets/detail/cancer (Updated 03 March 2021)
3. Yang JD et al. *Nat Rev Gastroenterol Hepatol* 2019;16(10):589–604
4. Marcon PdS et al. *World J Gastroenterol* 2018;24(5):613–622
5. Song et al. *Translational Gastrointestinal Cancer* 2014;3:103–105
6. Yu R, et al. *BMC Cancer* 2017;17:608
7. Gish RG. *Gastroenterol Hepatol* 2014;10:121–123.
8. Clopper CJ and Pearson ES. *Biometrika* 1934;404–413.

Acknowledgments

- This study was funded by Roche Diagnostics.
- Editorial support was provided by Nichola Cruickshanks of inScience Communications, Springer Healthcare (UK) and was funded by Roche Diagnostics.
- ELECSYS and COBAS are trademarks of Roche.

GET POSTER PDF

Access this poster using the Quick Response (QR) code above or by visiting https://bit.ly/3hBS9bo.

__DISCLOSURES:__ HL-YC reports consultancy fees from Abbvie, Arbutus Biopharma, Hepion, Gilead Sciences, Glaxo-Smith-Kline, GRAIL, MedImmune, Merck, Janssen Pharmaceutica, Intellia therapeutics, Roche, Vir Biotechnology, Aligos Therapeutics, Vaccitech and VenatoRx Pharmaceuticals and speaker's bureau participation for Gilead Sciences, Roche Diagnostics, and Mylan. AV reports consultancy fees and speaking and teaching for Roche; TB reports consultancy fees from Bayer, Eisai, Ipsen, Merck Sharp & Dome/Merck, and Roche; ENDT reports consultancy fees from AstraZeneca, Bayer, BMS, Eisai, Eli Lilly & Co, Pfizer, IPSEN and Roche, grant/research support from ArQule, AstraZeneca, BMS, Bayer, Celsion, Eli Lilly & Co and Roche and speaking and teaching for BMS and Falk; MK reports speaking and teaching for Eisai, Bayer, Merck Sharp & Dome, Bristol Myers Squibb, Eli Lilly & Co, EA Pharma, grant/research support from Gilead Sciences, Taiho, Sumitomo Dainippon Pharma, Takeda, Otsuka, EA Pharma, AbbVie, Eisai, Ono and advisory committee or review panel for Eisai, Ono, MSD, Bristol Myers Squibb and Roche; JT, reports consultancy fees for Amgen, Bayer Healthcare, Bristol Myers Squibb, Eisai, Ipsen, Merck Serono, Merck Sharp & Dome, Lilly ImClone and Roche; AE is an independent contractor for Roche Diagnostics; HGK and JKH have no conflicts to declare; KK, KM and AS are employees of Roche Diagnostics; TP reports speaker's bureau participation for Bristol-Myers Squibb, Gilead Science, Bayer, Abbott and Eisai and MSD and research grant/contracts from Gilead Science, Roche Diagnostics, Jannsen Fibrogen and VIR
