# Supplementary material for: Early-stage hepatocellular carcinoma screening in patients with chronic hepatitis B in China: a cost–effectiveness analysis
Source: J Comp Eff Res. 2024 Feb 28;13(4):e230146. doi: 10.57264/cer-2023-0146 (PMC11044951; doi:10.57264/cer-2023-0146)
Supplement: Supplementary file 2 [file cer-13-230146-s2.pdf]

Elecsys GAAD

cobas®

REF		SYSTEM
09342192001		**cobas e** 402<br>**cobas e** 411<br>**cobas e** 601<br>**cobas e** 602<br>**cobas e** 801

English

Please note

PRECAUTION: The Elecsys AFP and Elecsys PIVKA-II assays must be used for the Elecsys GAAD (**G**ender, **A**ge, **A**FP, **D**CP (PIVKA-II)) test. Assay results from other manufacturers have not been validated for the use of the Elecsys GAAD test. The Elecsys GAAD test should not be used without an independent clinical/radiological evaluation for the diagnosis of Hepatocellular Carcinoma (HCC).
For each Elecsys GAAD determination the measurement of the Elecsys AFP and Elecsys PIVKA-II assays must be determined from the same sample and measurement performed on the same analyzer type (either on **cobas e** 402, **cobas e** 411, **cobas e** 601, **cobas e** 602 or **cobas e** 801 analyzers).

Intended use

Elecsys GAAD is an in vitro diagnostic multivariate index assay intended to provide a semi-quantitative result by combining in an algorithm the quantitative measurements of Elecsys AFP assay and Elecsys PIVKA-II assay in human serum and plasma with gender and age.
Elecsys GAAD is intended as an aid in diagnosis of early stage Hepatocellular Carcinoma (HCC). Elecsys GAAD is indicated for adults who meet the following criteria: diagnosis of chronic liver disease and recommended for surveillance due to increased risk of developing HCC. Elecsys GAAD must be interpreted in conjunction with other diagnostic findings and clinical information in accordance with standard clinical management guidelines.

Summary

Liver cancer is the sixth most common cancer worldwide (excluding non-melanoma skin cancer) with over 905677 cases and the third most common cause of cancer death accounting for over 830180 deaths in 2020. With a ratio of mortality to incidence of > 0.92, the prognosis for patients who develop liver cancer is extremely poor.[1] HCC accounts for more than 90 % of primary liver cancer. Risk factors for HCC include chronic hepatitis B and hepatitis C, alcohol addiction, metabolic liver disease (particularly nonalcoholic fatty liver disease) and exposure to dietary toxins such as aflatoxins and aristolochic acid. All these risk factors are potentially preventable, highlighting the considerable potential of risk prevention for decreasing the global burden of HCC. HCC surveillance and early detection increase the chance of potentially curative treatment; however, HCC surveillance is substantially underutilized, even in countries with sufficient medical resources. Early-stage HCC can be treated curatively by local ablation, surgical resection or liver transplantation. Together, rational deployment of prevention, attainment of global goals for viral hepatitis eradication, and improvements in HCC surveillance and therapy hold promise for achieving a substantial reduction in the worldwide HCC burden within the next few decades.
The American Association for the Study of Liver Diseases (AASLD) guidelines recommend surveillance of high risk populations for HCC, every 6 months using ultrasound, either with or without a blood test to check protein levels (AFP).[2] Ultrasound examinations can be sensitive enough to detect small masses on the liver. While easily done in a doctor's office, ultrasound examinations are less conclusive with inexperienced technicians and in patients with obesity and fatty liver disease. A recent meta-analysis suggested that ultrasound may miss more than half of early stage HCCs.[3] Therefore, other methods of diagnosis, such as abdominal CT scan, abdominal MRI scan or liver biopsy are often needed.[2] These are more accurate than ultrasound, though are more invasive and uncomfortable for patients, as well as being costly to the healthcare system.

The European Association of Study of Liver (EASL) recommends that Surveillance should be performed by experienced personnel in all high-risk populations using abdominal ultrasound every six months (evidence moderate; recommendation strong). Ultrasound (US) is the method of choice and is often applied beyond HCC surveillance to monitor other conditions, such as the development of portal hypertension, including the onset of ascites or portal vein thrombosis.[4]

As per the Asian Pacific Association for the Study of the Liver (APASL) HCC Guidelines, surveillance for HCC should be undertaken in high risk groups of patients and is recommended (B2). The combination of US and serum AFP measurement performed biannually should be used as a surveillance strategy for HCC.[5] Measurement of serum alpha1-fetoprotein (AFP) levels is a commonly used strategy for surveillance of HCC because it is widely available, inexpensive, and easy to perform. However, AFP has suboptimal performance as a serological test for surveillance of HCC because it depicts fluctuating levels in patients with cirrhosis with a flare of HCV or HBV infection, in exacerbations of the underlying liver disease, or with the occurrence of HCC. PIVKA-II is a precursor and abnormal form of prothrombin that is found in patients with HCC. This can be used to differentiate HCC from non-HCC hepatic diseases. Alternate name for PIVKA-II is des-gamma-carboxy prothrombin (DCP).

The GAAD score can be easily calculated based on patient demographics and measurement of blood-based tumor markers using a small volume of blood draw. The GAAD score might be a practical, effective surveillance test where ultrasonography equipment and trained radiologists are scarce, which might improve compliance with surveillance and lead to improved effectiveness of cancer control programmes.[6]

The Elecsys GAAD test may be used in conjunction with diagnostic methods to detect early stage hepatocellular carcinoma.

Test principle

Refer to the respective Method Sheet of the Elecsys AFP and Elecsys PIVKA-II assays for assay-specific information.

Elecsys GAAD test

Elecsys GAAD is a semi-quantitative test for serum and plasma that combines the results of the Elecsys AFP and Elecsys PIVKA-II assays with gender and age.
Refer to the section Calculation of the Elecsys GAAD test using Elecsys assays.

Reagents - working solutions

Elecsys AFP assay

The reagent rackpack is labeled as AFP.

The **cobas e** pack is labeled as AFP.

Elecsys PIVKA-II assay

The reagent rackpack is labeled as PIVKA-II.

The **cobas e** pack is labeled as PIVKA-II.

Refer to the respective Method Sheet of the Elecsys AFP and Elecsys PIVKA-II assays for assay-specific information.

Reagent handling

Elecsys AFP and Elecsys PIVKA-II assays

The reagents in the respective kits have been assembled into a ready-for-use unit that cannot be separated.

All information required for correct operation is read in from the respective reagent barcodes.

Refer to the respective Method Sheet of the Elecsys AFP and Elecsys PIVKA-II assays for assay-specific information.

Storage and stability

Refer to the respective Method Sheets for reagent-specific storage and stability information.

Specimen collection and preparation

The Elecsys GAAD test using the Elecsys AFP and Elecsys PIVKA-II assays is intended for use with serum collected using standard sampling

tubes or tubes containing separating gel and plasma samples collected in Li-heparin as well as Li-heparin plasma tubes containing separating gel.

The Elecsys AFP and Elecsys PIVKA-II assays values must be determined from the same sample.
Refer to the respective Method Sheet of the Elecsys AFP and Elecsys PIVKA-II assays for assay-specific information.

Materials provided

Access to the Elecsys GAAD web calculator

Materials required (but not provided)

- REF 04481798190, Elecsys AFP, 100 tests
- REF 04491742190, Elecsys AFP, 200 tests
- REF 07026706190, Elecsys AFP, 300 tests
- REF 08333602190, Elecsys PIVKA-II, 100 tests
- REF 08333629190, Elecsys PIVKA-II, 300 tests
- An internet-enabled device (PC, Tablet or Mobile) that has the most recent versions of Chrome, Safari, Edge and Firefox web browser installed on it

Refer to the respective Method Sheet of the Elecsys AFP and Elecsys PIVKA-II assays for assay-specific required materials.

Calculation of the Elecsys GAAD test

The Elecsys GAAD test provides two interfaces to the users for the calculation of the Elecsys GAAD result.

1. Graphical User Interface (Elecsys GAAD website)

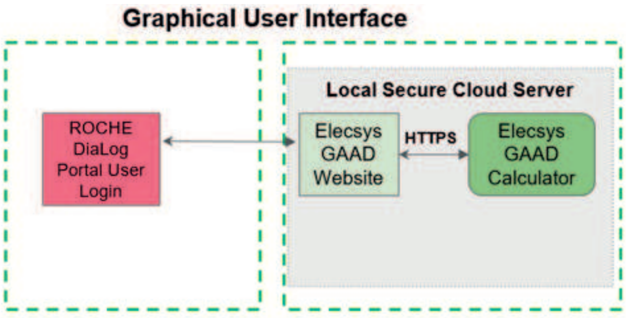

2. Laboratory Information System (LIS)/Electronic Medical Records (EMR) Interface

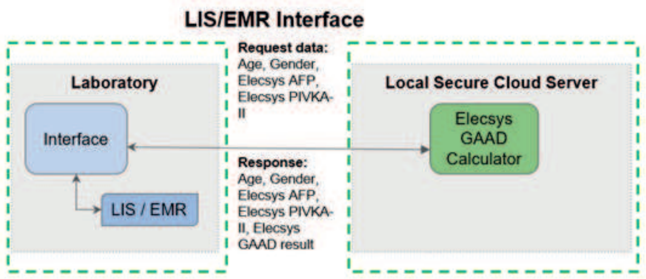

The LIS/EMR Interface will enable Laboratories to automatically trigger the calculation of the Elecsys GAAD result.
Through this interface, the Laboratories systems (LIS, EMR, Middleware) will be able to automatically send the following request data:

- Gender
- Age
- The Elecsys AFP assay value (for the range see section Calculation of the Elecsys GAAD using Elecsys assays, see also technical documentation)
- The Elecsys PIVKA-II assay value (for the range see section Calculation of the Elecsys GAAD using Elecsys assays, see also technical documentation)

The Elecsys GAAD calculator will respond with the Elecsys GAAD score, result including the input values.

How to use the Elecsys GAAD calculator

1. Access the Elecsys GAAD calculator by typing www.elecsys-gaad.com into your web browser.

2. You will be redirected to the DiaLog portal to login. Login or create a new account.

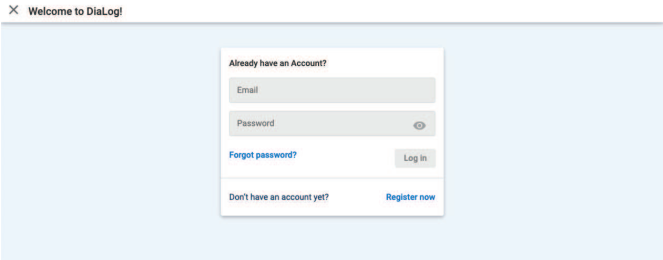

3. After logging in on the DiaLog portal, you will be redirected back to the Elecsys GAAD website.

4. A pop-up window appears with a "Terms of Service and Conditions of Use." Read the statement. Once the statement is understood, click on “I agree” to continue using the application.

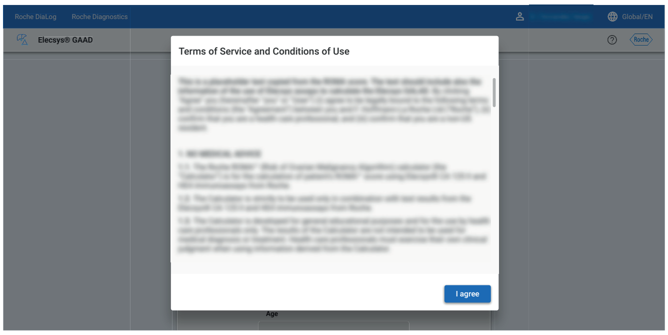

5. If “I agree” is chosen, the Elecsys GAAD calculator opens for use.

6. Enter the correct gender, age and Elecsys AFP assay value and Elecsys PIVKA-II assay value into the appropriate input fields. For age and test result enter only numerical data. Valid ranges are displayed in grey text under each input field. If non-numerical data or data out of range is entered, the input field will be marked red as an alert to correct the associated data entry error (see section Calculation of the Elecsys GAAD test using Elecsys assays).

Elecsys GAAD

cobas®

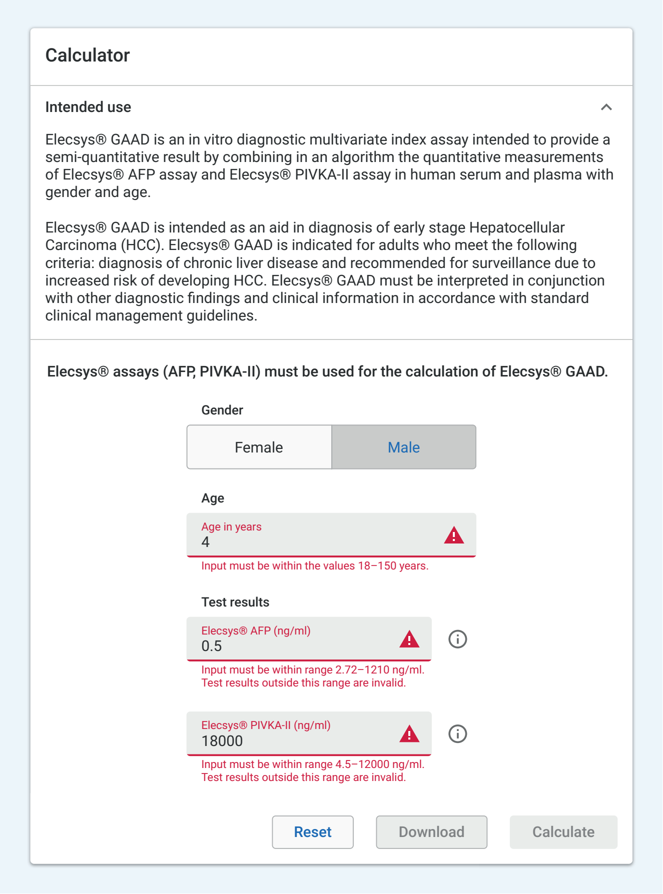

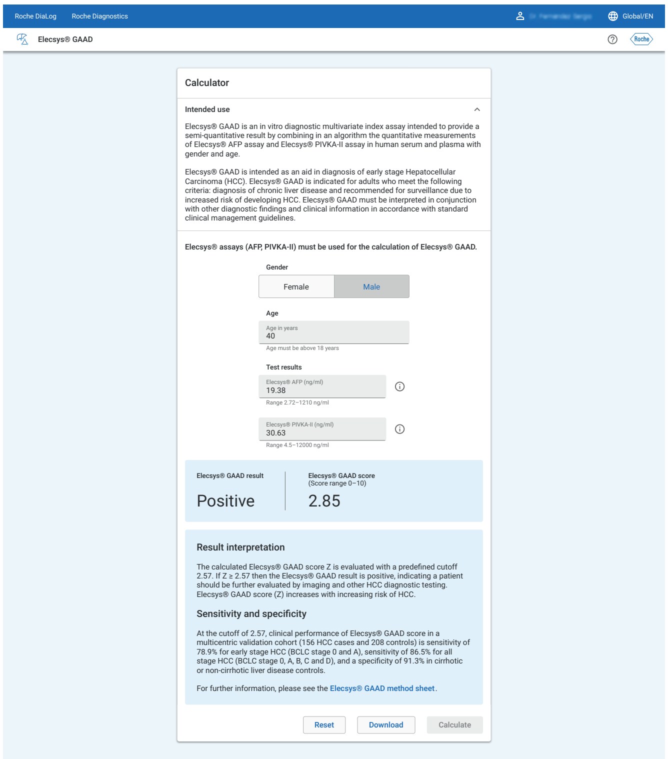

7. Once the data inputs are correctly entered, click on the “Calculate” button beneath the input fields.

8. Results from the calculations will be displayed in the results section of the screen:

- Inputs for gender, age, the Elecsys AFP and the Elecsys PIVKA-II assays will be verified and repeated.
- Elecsys GAAD score from 0-10.
- Positive/Negative Elecsys GAAD result.

9. Print or Download calculated results as needed.

10. To continue using the Elecsys GAAD calculator, overwrite the input values with new data values as instructed in step 6 and continue through steps 7, 8 and 9.

11. To close the Elecsys GAAD website, click on the X in the web browser.

12. To re-open and use the Elecsys GAAD calculator, go to Step 1.

Limitations of the Elecsys GAAD test

The Elecsys GAAD test uses the combination of gender, age, Elecsys AFP and Elecsys PIVKA-II assay results.

- The Elecsys GAAD result cannot be used as absolute evidence for the presence or absence of malignant disease.
- The Elecsys GAAD test should not be used as a standalone cancer screening test.
- The Elecsys GAAD test has only been evaluated in adults ≥ 18 years of age with a diagnosis of chronic liver disease and under surveillance due to increased risk of developing HCC and is only intended for use in this population.
- The Elecsys GAAD test should not be used without an independent clinical evaluation and is not intended to determine whether a patient should proceed to treatment. A negative Elecsys GAAD result, in the setting of a positive imaging examination should not preclude oncology referral.
- The Elecsys GAAD test has not been validated for the following groups: adults previously treated for malignancy, adults currently being treated for malignancy and children < 18 years of age.

The Elecsys AFP assay results should not be used interchangeably with other manufacturers’ methods for AFP determinations in the Elecsys GAAD calculator. Use only the Elecsys AFP assay values obtained from Roche.
The Elecsys PIVKA-II assay results should not be used interchangeably with other manufacturers’ methods for PIVKA-II or DCP determination in the Elecsys GAAD calculator. Use only the Elecsys PIVKA-II assay values obtained from Roche.
For the Elecsys AFP and Elecsys PIVKA-II assays input ranges see section Calculation of the Elecsys GAAD test using Elecsys assays.
Values obtained from non-designated methods or a mix of instrument platforms may produce incorrect Elecsys GAAD results. An error in the calculation of results could lead to inaccurate likelihood of malignancy assessment and improper management of the patient.
Refer to the respective Method Sheets of the Elecsys AFP and Elecsys PIVKA-II assays for assay-specific limitations and interferences.

cobas®

Early detection in patients with chronic liver disease

The effectiveness of the Elecsys GAAD test as an aid in diagnosing of early stage HCC was determined in a prospective/retrospective, multi-center, case-control, observational study with HCC cases (confirmed diagnosis) and with control subjects (clinical diagnosis of cirrhosis of any etiology, non-cirrhotic chronic HBV, non-cirrhotic chronic HCV and non-cirrhotic, non-alcoholic steatohepatitis (NASH).

Calculation of the Elecsys GAAD test using Elecsys assays

The Elecsys GAAD test is calculated based on gender (male or female), age in years and the results of Elecsys AFP and Elecsys PIVKA-II assays. Note: The equation used for the calculation of the Elecsys GAAD test uses values with the Elecsys AFP assay from 2.72-1210 ng/mL and with the Elecsys PIVKA-II assay from 4.5-12000 ng/mL.

- If Elecsys AFP assay value is below or equal to 2.72 ng/mL, then input 2.72 ng/mL.
- If Elecsys AFP assay value is above or equal to 1210 ng/mL, then input 1210 ng/mL.
- If Elecsys PIVKA-II assay value is below or equal to 4.5 ng/mL, then input 4.5 ng/mL.
- If Elecsys PIVKA-II assay value is above or equal to 12000 ng/mL, then input 12000 ng/mL.

Interpretation of Elecsys GAAD semi-quantitative test

The calculated Elecsys GAAD score Z is evaluated with a predefined cutoff 2.57. If $Z \geq 2.57$ then the Elecsys GAAD result is positive, indicating a patient should be further evaluated by imaging and other HCC diagnostic testing. Elecsys GAAD score (Z) increases with increasing risk of HCC.

Clinical study for performance determination

To examine whether the Elecsys GAAD test provides additional information when used in combination with independent clinical or radiological evaluation a clinical study was performed. This study was a prospective, multi-center, blinded trial, which included 470 patients with chronic liver disease, where 364 patients met the inclusion criteria.
Of the patients, 156 were diagnosed with HCC and 208 were found to be HCC free. The Elecsys GAAD test was determined using samples collected pre-diagnostic and values compared to the diagnoses are analyzed below:

	Median age	Male %	Etiology					
			Cirrhosis %	Hepatitis B %	Hepatitis C %	NASH %	ALD %	Others %
Control (N = 208)	53	126 (60.6 %)	79 (38.0)	72 (34.6)	27 (13.0)	30 (14.4)	0 (0.00)	0 (0.00)
HCC (N = 156)	64	130 (83.3 %)	130 (83.3)	11 (7.05)	3 (1.92)	1 (0.641)	2 (1.28)	9 (5.77)

Range of the Elecsys GAAD score in HCC cases compared to controls

The following table and graph show the range of the Elecsys GAAD score in samples from HCC patients staged according to the Barcelona clinic liver cancer classification (BCLC) compared to controls.

Disease stage	N	Min/Max	Median	25th-75th perc.*
Control	208	0.025/8.93	0.485	0.229-1.07
Early (Stage 0 and A)	71	0.322/10	6.94	3.27-9.14
BCLC Stage 0	10	0.741/7.64	3.36	-
BCLC Stage A	61	0.322/10	7.04	4.19-9.34
Late (Stage B, C, and D)	85	0.128/10	9.95	8.67-10
BCLC Stage B	26	1.37/10	9.36	5.23-9.94
BCLC Stage C	51	0.128/10	9.98	9.22-10
BCLC Stage D	8	9.99/10	10	-

* = not calculated for sample sizes smaller or equal to 20

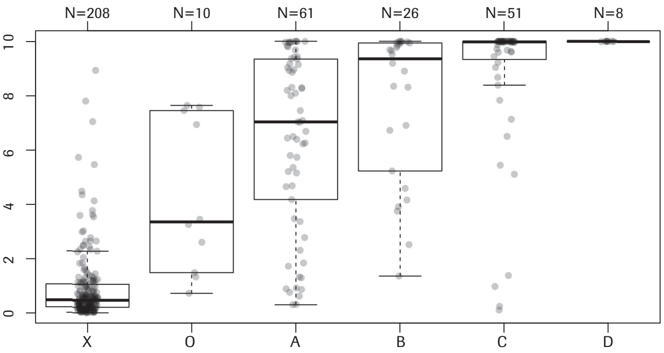

x --> X: Control; O: State 0; A: State A; B: State B; C: State C; D: State D
y --> Elecsys GAAD score

The Elecsys GAAD score and disease etiology

The following table and graph show the range of the Elecsys GAAD score in patient samples staged according to the etiology for both HCC cases (Label 1) and controls (Label 2).

Label (1 = Control, 2 = HCC)	Etiology	N	Min/Max	Median	25th-75th perc.*
1-A	Cirrhosis	79	0.041/8.93	0.78	0.401-1.85
1-B	Hepatitis B	72	0.025/5.73	0.357	0.152-0.627
1-C	Hepatitis C	27	0.044/1.85	0.415	0.229-0.943
1-D	NASH	30	0.059/3.63	0.447	0.251-0.696
1-E	ALD[a)]	0	-	-	-
1-F	Others	0	-	-	-
2-A	Cirrhosis	130	0.263/10	9	5.16-9.98
2-B	Hepatitis B	11	3.27/10	9.43	-
2-C	Hepatitis C	3	3.46/9.96	7.64	-
2-D	NASH	1	-	-	-
2-E	ALD	2	8.09/10	9.05	-
2-F	Others	9	0.128/10	9.97	-

a) Alcoholic liver disease

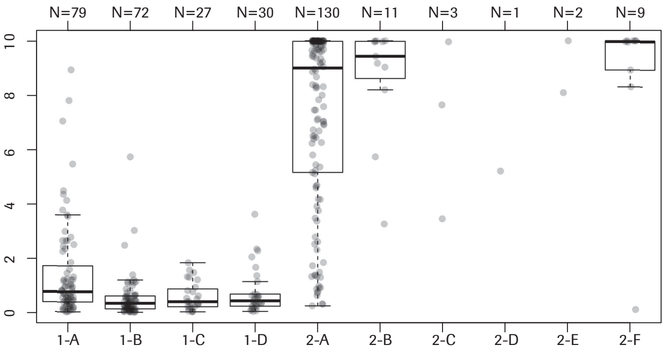

y --> Elecsys GAAD score

The Elecsys AFP assay and the Elecsys GAAD test summary by Cases and Controls, by region (Europe vs. Asia-Pacific) and all sites

			Control	HCC
		**N**	**208**	**156**
All Sites	Elecsys AFP (ng/mL)	Median	2.92	24.7
		Q25-Q75	2.72-4.71	5.94-528
		Q5-Q95	2.72-11.5	2.73-1210
		Min-Max	2.72-397	2.72-1210
		Missing (%)	0.0	0.0
	Elecsys GAAD	Median	0.485	9.16
		Q25-Q75	0.229-1.07	5.4-9.98
		Q5-Q95	0.06-3.6	0.881-10
		Min-Max	0.025-8.93	0.128-10
		Missing (%)	0.0	0.0
		N	112	87
Europe	Elecsys AFP (ng/mL)	Median	3.1	24.4
		Q25-Q75	2.72-5.22	6.22-687
		Q5-Q95	2.72-13.2	3.34-1210
		Min-Max	2.72-397	2.72-1210
		Missing (%)	0.0	0.0
	Elecsys GAAD	Median	0.563	9.67
		Q25-Q75	0.219-1.29	6.91-9.99
		Q5-Q95	0.057-4.49	1.34-10
		Min-Max	0.025-8.93	0.263-10
		Missing (%)	0.0	0.0
		N	96	69
Asia-Pacific	Elecsys AFP (ng/mL)	Median	2.82	25.8
		Q25-Q75	2.72-4.24	5.6-387
		Q5-Q95	2.72-11.5	2.73-1210
		Min-Max	2.72-80.9	2.72-1210
		Missing (%)	0.0	0.0
	Elecsys GAAD	Median	0.47	7.83
		Q25-Q75	0.23-0.76	3.46-9.95
		Q5-Q95	0.061-2.35	0.641-10
		Min-Max	0.043-4.14	0.128-10
		Missing (%)	0.0	0.0

Number of early stage HCC patients classified by the Elecsys AFP assay (at cutoff 20 ng/mL) vs. the Elecsys GAAD test (at cutoff 2.57)

Early stage HCC	Elecsys GAAD ≥ 2.57	Elecsys GAAD < 2.57	Total
AFP ≥ 20 (ng/mL)	27	0	27
AFP < 20 (ng/mL)	29	15	44
Total	56	15	71

Clinical performance of the Elecsys GAAD test for detection of early or late stage HCC at the cutoff 2.57

Parameter	Elecsys GAAD		
Staging	Early stage (Stage 0 and A)	Late stage (Stage B, C and D)	All stage
N (HCC)	71	85	156
N (total including control i.e. 208)	279	293	364
True Positives (TP)	56	79	135
True Negatives (TN)	190	190	190
False Positives (FP)	18	18	18
False Negatives (FN)	15	6	21
Sensitivity % (95% CI)[b]	78.9 (67.6, 87.7)	92.9 (85.3, 97.4)	86.5 (80.2, 91.5)
Specificity % (95% CI)	91.3 (86.7, 94.8)	91.3 (86.7, 94.8)	91.3 (86.7, 94.8)

b) Confidence interval

Clinical performance of the Elecsys GAAD test for the detection of early stage HCC for different etiologies at the cutoff 2.57

Parameter	Elecsys GAAD					
Etiology	Cirrhosis	HBV	HCV	NASH	ALD	Others
N (control; HCC)	133 (79;54)	80 (72;8)	30 (27;3)	31 (30;1)	1 (0;1)	4 (0;4)
TP	39	8	3	1	1	4
TN	64	70	27	29	0	0
FP	15	2	0	1	0	0
FN	15	0	0	0	0	0
Sensitivity % (95% CI)**	72.2 (58.4, 83.5)	–	–	–	-	-
Specificity % (95% CI)**	81 (70.6, 89)	97.2 (90.3, 99.7)	100 (87.2, 100)	96.7 (82.8, 99.9)	–	-

** = not calculated for sample sizes smaller or equal to 10

Cutoffs of the Elecsys GAAD test for early HCC at specified sensitivity or specificity

The following tables show the clinical performance of the Elecsys GAAD test at different cutoffs for specified sensitivity or specificity on all stage HCC or controls.

Specificity	Elecsys GAAD cutoff	Sensitivity (95 % CI) Early stage (Stage 0 and A)	Sensitivity (95 % CI) Late stage (Stage B, C and D)	Sensitivity (95 % CI) All stage	Specificity (95 % CI)
95 %	3.63	70.4 (58.4, 80.7)	92.9 (85.3, 97.4)	82.7 (75.8, 88.3)	95.2 (91.3, 97.7)
90 %	2.49	78.9 (67.6, 87.7)	94.1 (86.8, 98.1)	87.2 (80.9, 92)	90.4 (85.5, 94)
85 %	1.50	84.5 (74, 92)	94.1 (86.8, 98.1)	89.7 (83.9, 94)	85.1 (79.5, 89.6)
80 %	1.22	88.7 (79, 95)	96.5 (90, 99.3)	92.9 (87.7, 96.4)	80.3 (74.2, 85.5)
75 %	1.09	88.7 (79, 95)	96.5 (90, 99.3)	92.9 (87.7, 96.4)	75 (68.5, 80.7)
70 %	0.879	93 (84.3, 97.7)	97.6 (91.8, 99.7)	95.5 (91, 98.2)	70.2 (63.5, 76.3)

Sensitivity	Elecsys GAAD cutoff	Sensitivity (95 % CI) Early stage (Stage 0 and A)	Sensitivity (95 % CI) Late stage (Stage B, C and D)	Sensitivity (95 % CI) All stage	Specificity (95 % CI)
95 %	0.881	93 (84.3, 97.7)	97.6 (91.8, 99.7)	95.5 (91, 98.2)	70.7 (64, 76.8)
90 %	1.4	84.5 (74, 92)	95.3 (88.4, 98.7)	90.4 (84.6, 94.5)	83.7 (77.9, 88.4)
85 %	3.27	76.1 (64.5, 85.4)	92.9 (85.3, 97.4)	85.3 (78.7, 90.4)	94.2 (90.1, 97)
80 %	4.6	69 (56.9, 79.5)	89.4 (80.9, 95)	80.1 (73, 86.1)	97.6 (94.5, 99.2)
75 %	5.44	62 (49.7, 73.2)	85.9 (76.6, 92.5)	75 (67.4, 81.6)	97.6 (94.5, 99.2)
70 %	6.5	53.5 (41.3, 65.5)	84.7 (75.3, 91.6)	70.5 (62.7, 77.5)	98.6 (95.8, 99.7)

Cutoffs Receiver Operating Characteristic (ROC) for the Elecsys GAAD test and Elecsys AFP assay for early HCC

Area Under the Curve (AUC) values (95 % CI)): Elecsys AFP: 85.3 % (80.1 %-90.5 %), Elecsys GAAD: 92.9 % (89.4 %-96.4 %)

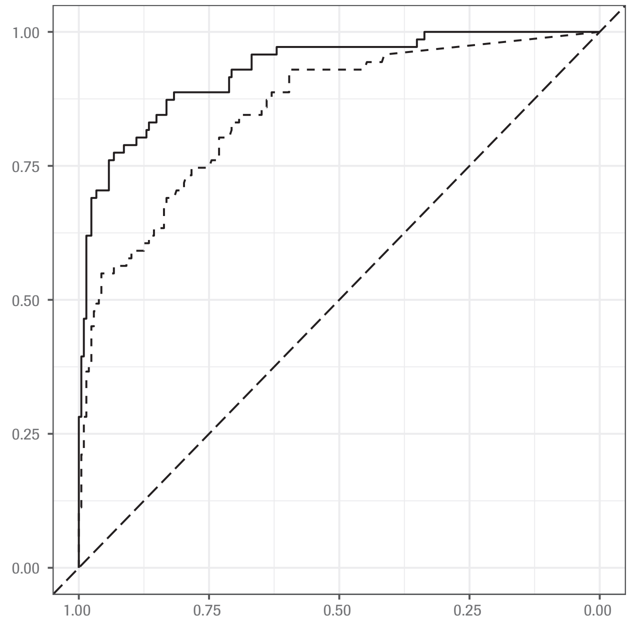

Solid line: the Elecsys GAAD test, dashed line: the Elecsys AFP assay, long dashed line: angle bisector
x --> specificity
y --> sensitivity

Number of all stage, early stage, late stage HCC cases, number of controls, and the HCC fraction for given Elecsys GAAD score ranges

Elecsys GAAD score range	**Number of subjects in GAAD score range**	**Number of early stage HCC**	**Number of late stage HCC**	**Number of all stage HCC**	**Number of controls**	**Early stage HCC proportion % (95 % CI)**	**All stage HCC proportion % (95 % CI)**
0 ≤ x < 2.57	211	15	6	21	190	7.11 (4.03, 11.5)	9.95 (6.27, 14.8)
2.57 ≤ x < 5.0	26	9	4	13	13	34.6 (17.2, 55.7)	50 (29.9, 70.1)
5.0 ≤ x < 7.5	27	17	7	24	3	63 (42.4, 80.6)	88.9 (70.8, 97.6)
7.5 ≤ x ≤ 10	100	30	68	98	2	30 (21.2, 40)	98 (93, 99.8)

Bar plot showing the fraction of all stage HCC cases within all subjects for different Elecsys GAAD score ranges, with the corresponding error bars (95 % CI)

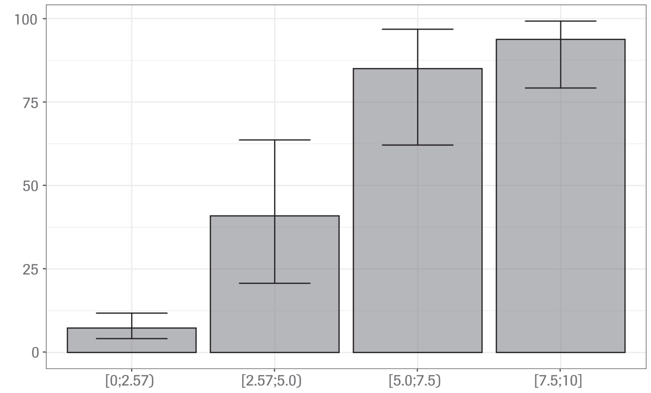

x --> The Elecsys GAAD score ranges, [a;b) denotes an open interval (a ≤ x < b) and [a;b] a closed interval (a ≤ x ≤ b)
y --> Proportion of all stage HCC within the Elecsys GAAD score ranges in %

References

1 Ferlay J, Soerjomataram I, Ervik M, et al. GLOBOCAN 2020, Cancer Incidence and Mortality Worldwide: IARC Lyon, France: International Agency for Research on Cancer; 2020. Available from: https://gco.iarc.fr/today/fact-sheets-cancers.

2 Marrero J, Kulik L, Sirlin C, et al. Diagnosis, staging, and management of hepatocellular carcinoma: 2018 practice guidance by the American Association for the Study of Liver Diseases. Hepatology. 2018 Aug;68(2):723-750.

3 El-Serag H. Epidemiology of viral hepatitis and hepatocellular carcinoma. Gastroenterology. 2012 May;142(6):1264-1273.

4 European Association for the Study of the Liver. Electronic address: easloffice@easloffice.eu; European Association for the Study of the Liver. EASL Clinical Practice Guidelines: Management of hepatocellular carcinoma. J Hepatol. 2018 Jul;69(1):182-236.

5 Omata M, Cheng A, Kokudo N, et al. Asia-Pacific Clinical Practice Guidelines on the Management of Hepatocellular Carcinoma: A 2017 Update. Hepatol Int. 2017 Jul;11(4):317-370.

6 Yang J, Roberts L. Hepatocellular carcinoma: a global view. Nat Rev Gastroenterol Hepatol. 2010 Aug;7(8):448-58.

For further information, please refer to the appropriate operator's manual for the analyzer concerned, the respective application sheets, the product information and the Method Sheets of all necessary components (if available in your country).

A point (period/stop) is always used in this Method Sheet as the decimal separator to mark the border between the integral and the fractional parts of a decimal numeral. Separators for thousands are not used.

Symbols

Roche Diagnostics uses the following symbols and signs in addition to those listed in the ISO 15223-1 standard (for USA: see dialog.roche.com for definition of symbols used):

CONTENT	Contents of kit

Elecsys GAAD

cobas®

SYSTEM	Analyzers/Instruments on which reagents can be used
REAGENT	Reagent
CALIBRATOR	Calibrator
⟶	Volume for reconstitution
GTIN	Global Trade Item Number

COBAS, COBAS E and ELECSYS are trademarks of Roche.
All other product names and trademarks are the property of their respective owners.

Additions, deletions or changes are indicated by a change bar in the margin.

© 2021, Roche Diagnostics

CE

Roche Diagnostics GmbH, Sandhofer Strasse 116, D-68305 Mannheim
www.roche.com

Roche
