# Supplementary material for: Early-stage hepatocellular carcinoma screening in patients with chronic hepatitis B in China: a cost–effectiveness analysis
Source: J Comp Eff Res. 2024 Feb 28;13(4):e230146. doi: 10.57264/cer-2023-0146 (PMC11044951; doi:10.57264/cer-2023-0146)
Supplement: Supplementary file 3 [file cer-13-230146-s3.pdf]

P161

The clinical utility of Elecsys GAAD score in the diagnosis of hepatocellular carcinoma

2022 APASL Single Topic Conference on Hepatocellular Carcinoma

Chung-Feng Huang[1], Ashish Sharma[2], Ming-Lung Yu[1]

[1]Hepatobiliary Division, Department of Internal Medicine, Kaohsiung Medical University Hospital, Kaohsiung Medical University, Kaohsiung, Taiwan

[2]Clinical Development and Medical Affairs, Roche Diagnostics International AG, Rotkreuz, Switzerland

Background

Regional guidelines have suggested sonography with or without adding serum tumor markers as the standard of hepatocellular carcinoma (HCC) surveillance. The performance of Elecsys® GAAD score, which includes the parameters of gender, age, alpha-fetoprotein (AFP) and protein induced by Vitamin K absence or antagonists-II (PIVKA-II*)* in the diagnosis of HCC is elusive.

Aims

The current study aims to address the diagnostic accuracy of HCC using Elecsys GAAD algorithmic score as compared to AFP and PIVKA-II with or without the complementation of sonography.

Methods

A total of 430 subjects including 200 healthy controls, 117 non-cirrhotic patients with chronic liver disease (chronic hepatitis B [CHB, n=67] and chronic hepatitis C [CHC, n=50]), 60 cirrhotic patients ([CHB, n=11] and [CHC, n=49]) and 53 HCC ([CHB, n=33], [CHC, n=19] and [non CHB/CHC, n=1]) were enrolled. Sonography was performed every 6 months in patients with liver disease. Blood sampling was collected within 3 months before HCC diagnosis in the 53 HCC patients.

Results

Of the 53 HCC patients, the distribution of Barcelona Clinic Liver Cancer (BCLC) classification was BCLC 0 (n=7, 13.2%), BCLC A (n=19, 35.8%), BCLC B (n=12, 22.6%) and BCLC C (n=15, 28.3%), respectively. By adopting the clinically validated Elecsys GAAD cut-off value of 2.57, the sensitivity of AFP, PIVKA-II and GAAD in the diagnosis of HCC was 43.4%, 69.8% and 77.4%, respectively, whereas the area under ROC (AUROC) was 0.79, 0.92 and 0.92, respectively. Notably, GAAD has a substantially better sensitivity than PIVKA-II in HCC patients with very early stage (BCLC 0, 57.1% vs. 26.6%). While comparing the different performances between PIVKA-II and GAAD among the 26 patients with early HCC (BCLC 0-A), there was only one patient who had elevated PIVKA-II but with a normal GAAD score. By contrast, 4 early HCC patients had normal PIVKA-II but presented with abnormal GAAD scores. By the complementation of sonography, the sensitivity of GAAD was 92.5%, and reached up to 100% in the subgroups of BCLC C, non-cirrhotic as well as CHC patients.

Conclusions

PIVKA-II and GAAD are more sensitive than AFP in HCC diagnosis. Elecsys GAAD score is highly accurate in the diagnosis of HCC, which may outperform PIVKA-II in particular in patients with early HCC. The complementation with sonography further added value for HCC diagnosis.

	Healthy control (n=200)	CHC/non-LC (n=50)	CHB/non-LC (n=67)	CHC/LC (n=49)	CHB/LC (n=11)	HCC* (n=53)
Male gender, n (%)	53 (26.5)	25 (50.0)	45 (67.2)	23 (46.9)	7 (63.6)	40 (75.5)
Age, yrs (mean±SD)	54.4±15.9	58.4±13.1	56.0±13.0	66.4±11.2	58.7±9.0	64.1±9.9
Platelet count, x1000 /mm³ (mean±SD)	263±64	198±59	207±90	144±66	115±49	192±94
AST, IU/L (mean±SD)	20±5	51±32	255±527	54±35	1007±926	66.8±77.2
ALT, IU/L (mean±SD)	18±7	70±51	372±965	55±46	1201±992	48.7±62.1
AFP, ng/ ml, (mean±SD)	2.8±1.7	4.3±3.8	6.3±10.0	5.1±5.5	15.6±23.0	2808±10788
AFP, ng/ ml, (median, range)	2.4 (0.6-13.8)	3.1 (0.6-19.0)	3.0 (0.8-56.1)	3.4 (0.6-25.5)	7.5 (1.3-79.4)	10.2 (1.5-60138)
PIVKA-II, ng/mL, (mean±SD)	16.6±2.8	16.9±4.7	21.8±38.8	31.9±50.2	34.1±70.1	898±2077
PIVKA-II, ng/mL (median, range)	16.5 (10.3-33.2)	15.9 (11.5-41.4)	15.6 (3.8-316.9)	16.9 (10-319.6)	14.3 (7.4-245.4)	108.4 (13.2-10108)
GAAD score (mean±SD)	0.45±0.59	0.79±0.72	0.97±1.30	1.50±1.74	1.78±1.83	6.06±3.71
GAAD score (median, range)	0.23 (0.01-3.41)	0.59 (0.03-3.3)	0.47 (0.05-6.33)	0.97 (0.11-8.34)	0.90 (0.11-4.79)	6.6 (0.14-10.0)

Note: CHC: chronic hepatitis C. CHB: chronic hepatitis B. LC: liver cirrhosis. HCC: hepatocellular carcinoma. AFP: alpha fetoprotein. PIVKA-II: Protein induced by Vitamin K absence or antagonists-*II. SD: standard deviation.* *blood testing within 6 months before HCC occurrence

Characteristics of the 53 HCC patients

Male gender, n (%)	40 (75.5)
Age, yrs (mean±SD)	64.1±9.9
Body mass index, kg/m² (mean±SD)	25.2±3.5
Platelet count, x1000 /mm³ (mean±SD)	192±94
AST, IU/L (mean±SD)	66.8±77.2
ALT, IU/L (mean±SD)	48.7±62.1
Total bilirubin, mg/dL (mean±SD)	1.10±0.70
**Liver cirrhosis, n (%)**	32 (60.4)
Child-Pugh A, n (%)	25 (47.2)
Child-Pugh B, n (%)	7 (13.2)
Etiology	
HBV, n (%)	33 (62.3)
HCV, n (%)	19 (35.8)
NBNC, n (%)	1 (1.9)
BCLC stage	
BCBC 0, n (%)	7 (13.2)
BCLC A, n (%)	19 (35.8)
BCLC B, n (%)	12 (22.6)
BCLC C, n (%)	15 (28.3)
Largest tumor size, cm (mean±SD)	5.0±3.6
AFP, ng/ ml, (mean±SD)	2808±10788
AFP> 20 ng/ml, n (%)	23 (43.4)
PIVKA-II, ng/mL, (mean±SD)	898±2077
PIVKA> 28.4 ng/mL, n (%)	37 (69.8)
GAAD score, (mean±SD)	6.06±3.71
GAAD score >2.57, n (%)	41 (77.4)

AUROC

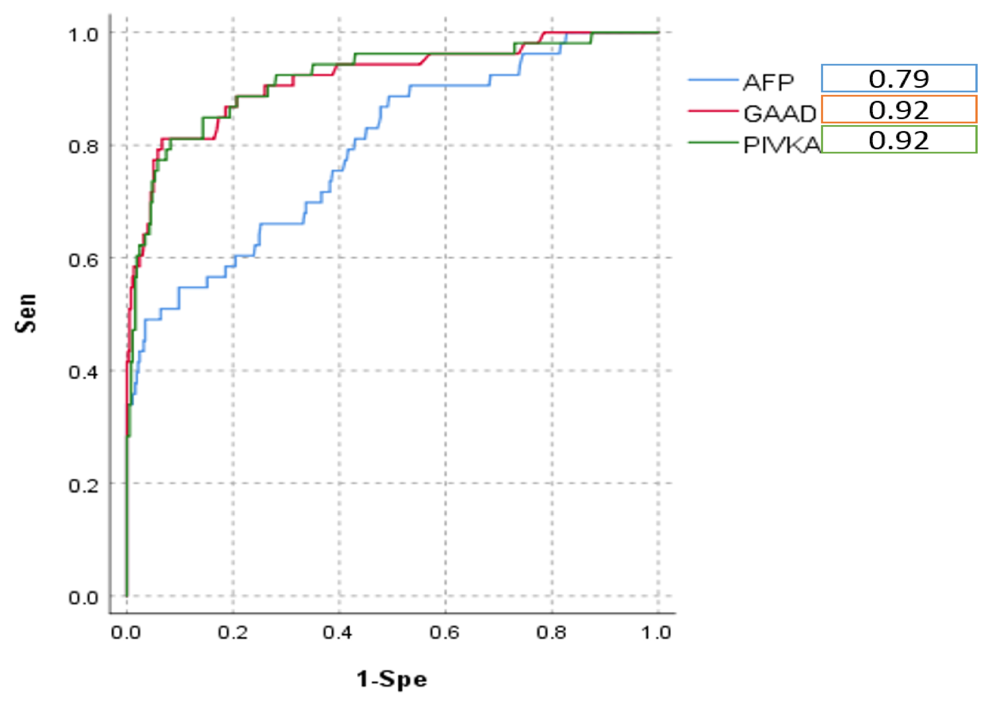

Cutoff value: AFP-20 ng/mL, PIVKA-II 28.4 ng/mL, GAAD 2.57

Sensitivity of the biomarkers and US

Cutoff value: AFP-20 ng/mL, PIVKA-II 28.4 ng/mL, GAAD 2.57

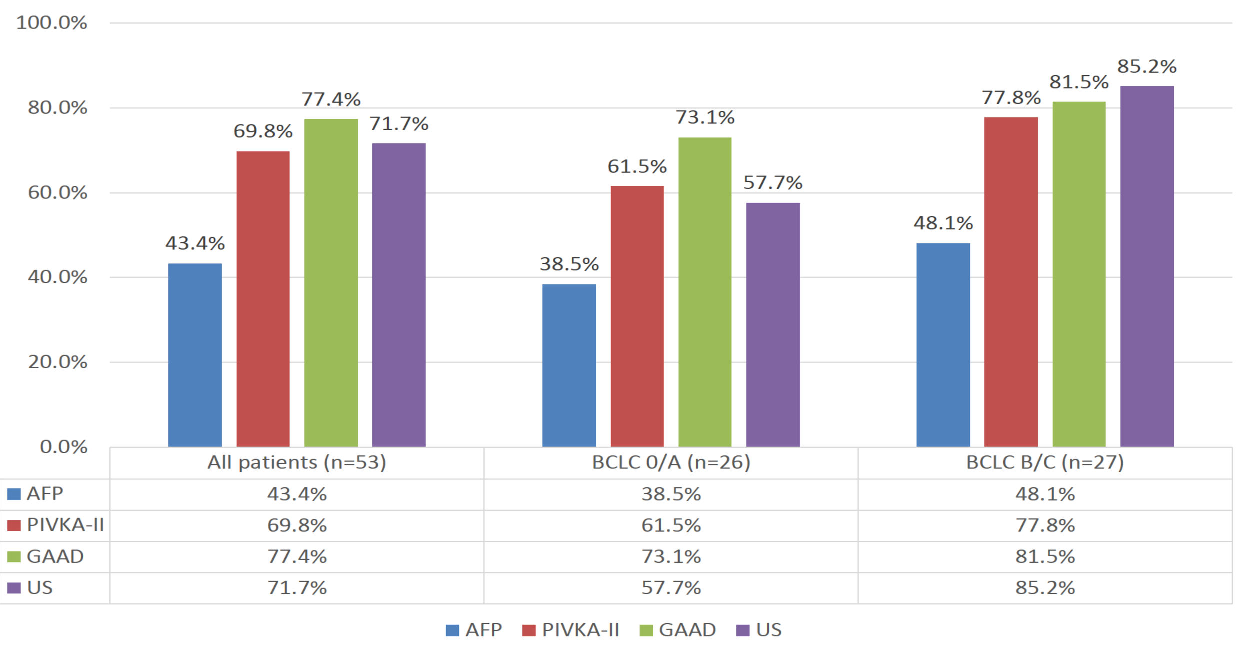

GAAD adds SEN to US in detecting early HCC

BCLC 0	US +	US -
GAAD >	0	4
GAAD ≤	2	1

BCLC A	US +	US -
GAAD >	11	4
GAAD ≤	2	2

BCLC B	US +	US -
GAAD >	8	1
GAAD ≤	2	1

BCLC C	US +	US -
GAAD >	11	2
GAAD ≤	2	0

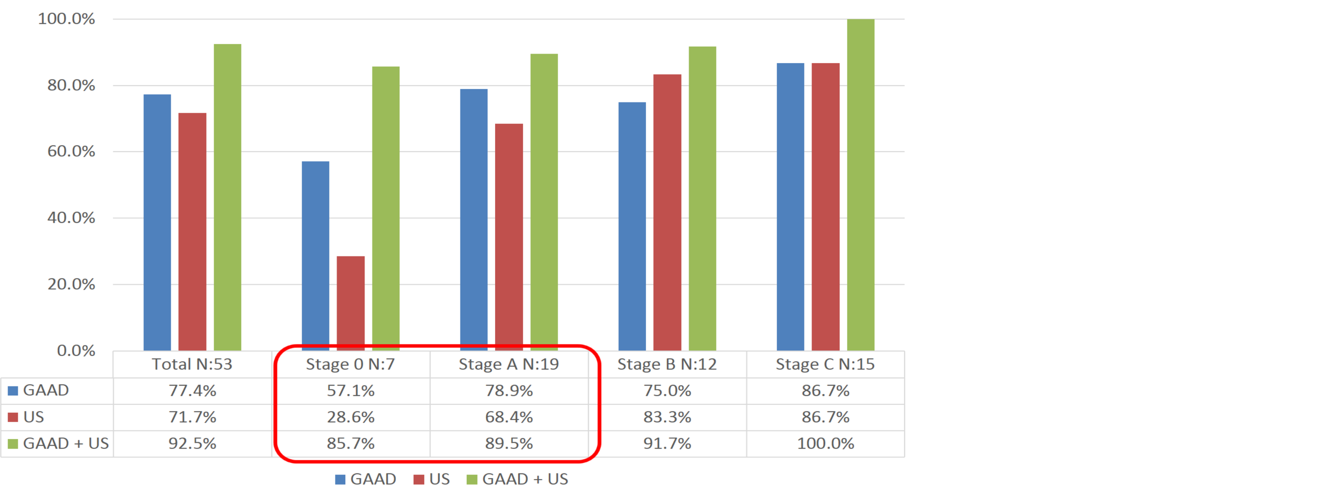

Comparison of GAAD and other markers and US in HCC patients

GAAD score serves a complementary tool with sonography in the diagnosis of HCC

US+GAAD sensitivity = 92.5% — All stage N=53

	US +	US -
GAAD >	30	11
GAAD ≤	8	4

	AFP >	AFP ≤
GAAD >	22	19
GAAD ≤	1	11

	PIVKA-II >	PIVKA-II ≤
GAAD >	34	7
GAAD ≤	3	9

US+GAAD sensitivity = 88.5% — Early stage N=26

	US +	US -
GAAD >	11	8
GAAD ≤	4	3

	AFP >	AFP ≤
GAAD >	9	10
GAAD ≤	1	6

	PIVKA-II >	PIVKA-II ≤
GAAD >	15	4
GAAD ≤	1	6

US+GAAD sensitivity = 96.3% — Late stage N=27

	US +	US -
GAAD >	19	3
GAAD ≤	4	1

	AFP >	AFP ≤
GAAD >	13	9
GAAD ≤	0	5

	PIVKA-II >	PIVKA-II ≤
GAAD >	19	3
GAAD ≤	2	3

Sensitivity/Specificity stratified by cirrhotic status

	Non-HCC, n (%)	HCC, n (%)	AUROC	P value	SEN (%)	SPE (%)	PPV (%)	NPV (%)	ACC (%)
**Non-LC**									
AFP> 20 ng/ml	5 (1.6)	6 (28.6)	0.78	<0.001	28.6	98.4	54.5	95.4	94.1
PIVKA-II> 28.4 ng/mL	6 (1.9)	14 (66.7)	0.94	<0.001	66.7	98.1	70.0	97.8	96.1
GAAD score >2.57	10 (3.2)	15 (71.4)	0.87	<0.001	71.4	96.8	60.0	98.1	95.2
GAAD score >2.57 plus sonography	10 (3.2)	21 (100)		<0.001	100	96.8	67.7	100	97.0
**LC**									
AFP> 20 ng/ml	5 (8.3)	17 (53.1)	0.76	<0.001	53.1	91.7	77.3	78.6	78.3
PIVKA-II> 28.4 ng/mL	12 (20.0)	23 (71.9)	0.87	<0.001	71.9	80.0	65.7	84.2	77.2
GAAD score >2.57	10 (16.7)	26 (81.3)	0.90	<0.001	81.3	83.3	72.2	89.3	82.6
GAAD score >2.57 plus sonography	10 (16.7)	28 (87.5)		<0.001	87.5	83.3	73.7	92.6	84.7

Note: HCC: hepatocellular carcinoma. AUROC: Area Under the Receiver Operating Characteristic curve. SEN: sensitivity. SPE, specificity. PPV: positive predictive value. NPV: neg predictive value. ACC: accuracy.
*Patient distribution in terms of BCLC stages (BCLC 0-A vs. BCLC B-C) was similar between cirrhosis and non-cirrhosis.

Sensitivity/Specificity stratified by etiology

	Non-HCC, n (%)	HCC, n (%)	AUROC	P value	SEN (%)	SPE (%)	PPV (%)	NPV (%)	ACC (%)
**HBV**									
AFP> 20 ng/ml	7 (9.0)	16 (48.5)	0.75	<0.001	48.5	91.0	69.6	80.7	78.4
PIVKA-II> 28.4 ng/mL	5 (6.4)	21 (63.6)	0.92	<0.001	63.6	93.6	80.8	85.9	84.7
GAAD score >2.57	9 (11.5)	24 (72.7)	0.88	<0.001	72.7	88.5	72.7	88.5	83.8
GAAD score >2.57 plus sonography	9 (11.5)	29 (87.9)		<0.001	87.9	88.5	76.3	94.5	88.3
**HCV**									
AFP> 20 ng/ml	3 (3.0)	7 (36.8)	0.75	<0.001	36.8	97.0	70.0	88.9	87.3
PIVKA-II> 28.4 ng/mL	12 (12.1)	15 (78.9)	0.89	<0.001	78.9	87.9	55.6	95.6	86.4
GAAD score >2.57	8 (8.1)	16 (84.2)	0.90	<0.001	84.2	91.9	66.7	96.8	90.7
GAAD score >2.57 plus sonography	8 (8.1)	19 (100)		<0.001	100	91.9	70.4	100	93.2

Note: HCC: hepatocellular carcinoma. AUROC: Area Under the Receiver Operating Characteristic curve. SEN: sensitivity. SPE, specificity. PPV: positive predictive value. NPV: negative predictive value. ACC: accuracy.
*Patient distribution in terms of BCLC stages (BCLC 0-A vs. BCLC B-C) was similar between viral etiology.

ELECSYS is a trademark of Roche.
